# Bent conformation of a backbone pilin N-terminal domain supports a three-stage pilus assembly mechanism

**DOI:** 10.1038/s42003-018-0100-0

**Published:** 2018-07-17

**Authors:** Priyanka Chaurasia, Shivendra Pratap, Airi Palva, Ingemar von Ossowski, Vengadesan Krishnan

**Affiliations:** 1 Laboratory of Structural Microbiology, Regional Centre for Biotechnology, NCR Biotech Science Cluster, Faridabad, 121001 India; 2 Department of Biotechnology, Manipal University, Manipal, Karnataka 576104 India; 3 Department of Veterinary Biosciences, University of Helsinki, FIN-00014 Helsinki, Finland

## Abstract

Effective colonization of host cells by some Gram-positive bacteria often involves using lengthy, adhesive macromolecular structures called sortase-dependent pili. Among commensals, the gut-adapted *Lactobacillus rhamnosus* GG strain encodes the operons for two varieties of these pili (SpaCBA and SpaFED), with each structure consisting of backbone, tip, and basal pilin subunits. Although the tertiary structure was recently solved for the backbone subunit (SpaA) of the SpaCBA pilus, no structural information exists for its counterpart in the SpaFED pilus. Here, we report several crystal structures for the SpaD backbone pilin, two of which capture the N-terminal domain in either the closed (linear) or open (bent) conformation. To our knowledge, this is the first observation of the bent conformation in Gram-positive pilin structures. Based on this bent conformation, we suggest a three-stage model, which we call the expose-ligate-seal mechanism, for the docking and assembly of backbone pilins into the sortase-dependent pilus.

## Abstract (web summary)

Priyanka Chaurasia et al. report crystal structures of the SpaD backbone pilin from a gut-adapted bacteria, *Lactobacillus rhamnosus*. The observed bent conformation of the N-terminal domain has not been seen in other Gram-positive pilin structures.

## Introduction

A key contributing factor of bacterial survival is the ability to colonize a host effectively. Among the tools used by bacteria for host-colonization are the easily-recognizable multi-subunit pili^1,2^. These pilus structures are lengthy and limb-like, and often have an adhesive character that fastens readily to cellular surfaces. Moreover, as a direct result of their long and extended peripheral reach, pili facilitate the first interactive contact that some bacteria have with the immediate environment. Although surface pili are not a universal trait of all bacteria, Gram-negative bacteria contain at least five types: chaperone-usher pili; type IV pili; conjugative type IV secretion pili; curli fibers; and type V pili^3,4^. In contrast, only two types are known to exist in Gram-positive bacteria: type IV pili^3,5^ and sortase-dependent pili^6^. The presence of sortase-dependent pili was first established among pathogenic species^6–8^, but more recently this form of piliation has been identified in gut commensals^9–11^. Since sortase-dependent pili are known to adhere to epithelial extracellular matrix proteins and mucus, this enables them to have an important role during host cell colonization as mechanisms of pathogenic virulence and non-pathogenic niche adaptation^6,12–14^.

The sortase-dependent pilus structure is usually built from three types of pilin subunits, each with a defined location and purpose. So-called major pilins polymerize head-to-tail into the pilus backbone, with ancillary pilins added at the tip (adhesin) and base (anchor). Typically, these backbone, tip, and basal subunits are held together by covalent bonds catalyzed by a pilin-specific C-type sortase enzyme. According to prevailing models^15^, pilus assembly begins when the sortase severs the threonine-glycine bond within the C-terminal LPXTG sorting motif of a tip pilin. This allows the freed threonine to form an isopeptide bond with the lysine of the YPKN pilin motif in the N-terminal domain of a backbone subunit. This process continues with a succession of backbone pilins, until the pilus structure reaches an adequate length, at which point further polymerization is halted. The arrival of a basal pilin attached to the housekeeping A-type sortase is the signal that initiates termination and anchoring activities^16^, whereby a C-type sortase catalyzes an isopeptide bond between the basal subunit and the backbone pilin most recently added^17,18^. The fully assembled pilus (carried by the A-type sortase) is then anchored to the bacterial cell wall when the LPXTG-motif threonine of the basal pilin covalently attaches to the peptidoglycan layer^17,18^. The genes for the backbone, tip, and basal pilins and the C-type sortase are encoded as a pilus operon in the genome, facilitating the coordinated expression of the various proteins involved^19^.

Based on their tertiary structures, backbone, tip, and basal pilins share a common structural topology^20–22^ by being comprised of the CnaA and CnaB domains. Both domains are variants of the immunoglobulin (Ig)-like domain fold that was first established in the staphylococcal collagen adhesin (Cna) protein^23–25^. The topological core of the CnaA and CnaB domains consists of nine and seven β-strands, respectively, and are distinguished by interspersed regions of loops, α-helices, β-strands, and additional domains. In the CnaA and CnaB domains, an autocatalytic internal isopeptide bond can occur between either lysine and aspartate (K–D) or lysine and asparagine (K–N), thus bolstering the structural rigidity of the pilin^26,27^. Most solved structures of the three pilin types are for those that comprise the pilus backbone^21^. Backbone pilins^27–41^ consist of between two to four of the CnaA and CnaB domains^21^, though the domain at the N-terminal region has some unique properties worth studying.

Recently, we reported the X-ray structural determination of double-domained SpaA, the backbone pilin from the *Lactobacillus rhamnosus* GG strain^29^. *L. rhamnosus* GG is a gut-adapted lactic acid bacterium best known for its advocated use as a probiotic^42^, but also for being identified as the first Gram-positive commensal capable of producing sortase-dependent pili (called SpaCBA)^9^. The SpaCBA pilus, which is encoded by the *spaCBA* operon and consists of the tip SpaC, basal SpaB, and backbone SpaA pilins, has undergone extensive functional and structural characterization over the past few years, and stems from a strong curiosity in unraveling the molecular aspects of *L. rhamnosus* GG gut ecology and probiosis^14^. The *L. rhamnosus* GG genome also carries the genes for another pilus operon called *spaFED*^9^. However, unlike the genes for the *L. rhamnosus* GG SpaCBA pilus, those for the SpaFED pilus are constitutively inactive under testing conditions^43^, though their expression might still rely on an unknown stimulus or else be gut-induced in situ^44^. While the native form of a fully assembled SpaFED pilus has to date only been predicted in *L. rhamnosus* GG^43^, the *spaFED* operon represents an intact translational unit whose cloned expression can be induced in *Lactococcus lactis* to produce a recombinant version consisting of the tip SpaF, basal SpaE, and backbone SpaD pilins^45^. To address what structural similarities or differences might exist between the two *L. rhamnosus* GG pilus types, we sought to solve the tertiary structures of the SpaCBA and SpaFED pilus proteins through X-ray crystallography^29,46–48^. In this present study, we bring new mechanistic insights about the pilin-assembly process for sortase-dependent pili by presenting crystal structures of the SpaD backbone pilin with its N-domain in both a closed (linear) and open (bent) conformation.

## Results

### Overall structure of GG-SpaD

**Fig. 1:**
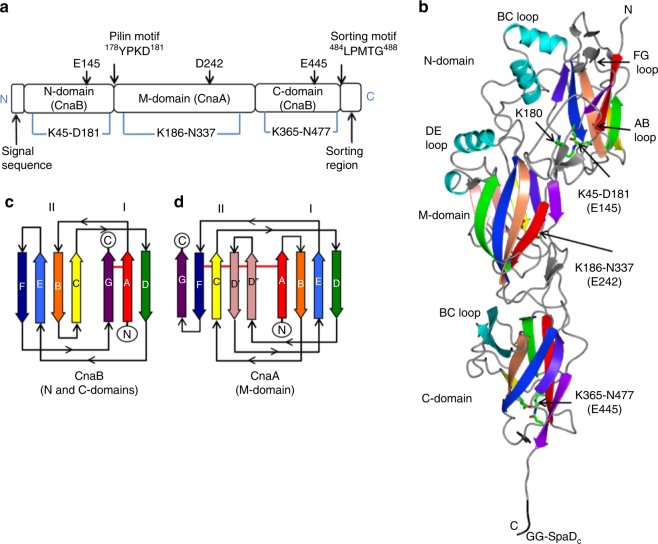
Domain and structural features of GG-SpaD. **a** Schematic diagram of GG-SpaD showing the domain boundaries and functional elements obtained from sequence and structural analysis. GG-SpaD is comprised of three structural domains, which includes the N-domain (36–183) with a CnaB fold, M-domain (184–358) with a CnaA fold, and C-domain (359–479) with a CnaB fold. Locations for the N-terminal secretion signal and C-terminal LPMTG-motif sorting region are indicated. The location of the YPKD pilin motif (which contains the linking lysine K180) is indicated and found between the N- and M-domains. Positions of the internal isopeptide bonds (at bottom) and their respective catalytic residues (at top) are marked. **b** Ribbon diagram depicting the crystal structure of GG-SpaD in the closed (linear) conformation (GG-SpaD_C_). The core β-strands of the N-, M-, and C-domains are indicated using rainbow colors. The linking lysine (K180) and the residues for internal isopeptide bonds are shown in sticks and identified by a black arrow. The C-terminal tail containing a partial sorting motif (in black) is shown protruding from the C-domain. Other secondary structures not part of the core fold are shown in pink and cyan. **c** Topology diagram of the CnaB fold (N- and C-domains). The core β-strands of two β-sheets are labeled A to G using rainbow colors. Horizontal red lines indicate the location of internal isopeptide bonds. **d** Topology diagram of the CnaA fold (M-domain). The core β-strands and the location of isopeptide bonds are indicated as in **c**. Additional β-strands not part of the typical Ig-like fold are shown in pink

In its unprocessed form, GG-SpaD (prefixed to avoid name overlap with *Corynebacterium diphtheriae* SpaD^33^) is a 517-residue protein (UniprotKB ID: A0A179XFF5) (Fig. 1a). To crystallize GG-SpaD, we produced a recombinant form using residues 36–485^46^. The tertiary organization of GG-SpaD is a tandem assembly of three Ig-like domains, wherein a middle domain (M-domain) joins together the N- and C-terminal regions (N- and C-domains) of the pilin subunit (Fig. 1a, b). The crystal structure of GG-SpaD_MC_, which contains just the M- and C-domains, was initially obtained at resolution 1.5 Å. The full-length structure containing all three domains was later determined from orthorhombic and hexagonal crystals, which yielded two distinct conformations, respectively, called the closed and open states (Table 1).

**Table 1:** Data collection and refinement statistics

	GG-SpaD_C_ (5YU5)	GG-SpaD_O_ (5YXO)	GG-SpaD_MC_ (5YXG)	GG-SpaD_D242A_ (5Z0Z)	GG-SpaD_K365A_ (5Z24)
Data collection					
Space group	*P* 2_1_ 2_1_ 2_1_	*P* 6_5_ 2 2	*P* 2_1_ 2_1_ 2_1_	*P* 2_1_ 2_1_ 2_1_	*P* 2_1_ 2_1_ 2_1_
Cell dimensions					
* a*, *b*, *c* (Å)	54.41, 76.26, 400.73	73.72, 73.72, 428.66	50.11, 83.16, 149.40	48.11, 75.51, 402.24	50.82, 74.89, 403.65
α,*β*,*γ* (°)	90, 90, 90	90, 90, 120	90, 90, 90	90, 90, 90	90, 90, 90
Resolution range (Å)	74.92–2.27 (2.54–2.27)	63.84–2.51 (2.71–2.51)	72.66–1.48 (1.62–1.48)	74.21–2.47 (2.87– 2.47)	45.68–2.40 (2.56–2.40)
Ellipsoidal resolution (Å) [direction]	3.30 [a*], 3.11 [b*], 1.89 [c*]	2.50 [0.89a*-0.44b*], 2.50 [b*], 2.95 [c*]	-	3.55 [a*], 4.12 [b*], 2.34 [c*]	3.71 [a*], 2.78 [b*], 2.04 [c*]
* R*_merge_	0.05 (0.47)	0.14 (0.82)	0.06 (0.54)	0.16 (1.01)	0.05 (0.43)
* I/σ*(*I*)	18.1 (2.7)	10.6 (1.4)	15.6 (3.2)	8.6 (1.9)	21.2 (3.6)
* CC*_1/2_	0.99 (0.93)	0.99 (0.28)	0.99 (0.81)	0.99 (0.66)	0.99 (0.96)
Completeness (%)	87.1 (48.5)	89.6 (45.5)	99.8 (99.8)	88.4 (57.5)	91.5 (80.8)
Redundancy	5.9 (4.5)	9.1 (3.6)	5.3 (5.2)	9.8 (6.7)	7.0 (5.2)
Refinement					
Resolution (Å)	2.3	2.5	1.5	2.5	2.4
No. reflections	39,269 (1964)	19,138 (957)	104,530 (24,710)	21,504 (1076)	35,611 (1782)
* R*_work_ / *R*_free_	0.201/0.236	0.243/0.291	0.144/0.186	0.225/0.261	0.242/0.278
No. atoms					
Protein	6864	3189	4646	6868	6771
Ion (Cl^-^)	0	0	4	0	0
Water	360	24	805	128	227
B-factors					
Protein	52.0	66.3	16.3	49.9	54.5
Ligand/ion	-	-	22	-	-
Water	32.3	49.7	36.9	43.8	33.3
R.m.s. deviations					
Bond lengths (Å)	0.007	0.006	0.001	0.007	0.008
Bond angles (°)	1.203	1.134	1.111	1.171	1.250

Each dataset was collected from a single crystal. Data values for the highest-resolution shell are in parentheses. Excluding the data for GG-SpaD_MC_, refinement values are after anisotropic correction. The resolution limits for three directions in reciprocal space from the analysis of anisotropy are calculated via the STARANISO server

The N- and C-domains (residues 36–183 and 359–479, respectively) both consist of four and three-stranded β-sheets and resemble the core fold of the CnaB domain (Fig. 1c). The M-domain (residues 184–358) comprises five and four-stranded β-sheets and is similar to the CnaA-type fold (Fig. 1d). A C-terminal tail (residues 480–485) projects from the C-domain core and contains the first two residues of the LPXTG motif (Fig. 1b). In the orthorhombic crystal the three domains are arranged linearly with an elongated shape 125 Å in length. This we call GG-SpaD_C_ for the closed conformational state. The M- and C-domains are both stabilized by an internal K–N isopeptide bond, normally found in most Gram-positive backbone pilins. The N-domain shows the atypical K–D isopeptide bond, involving the uncommon YPKD pilin motif. The two elongated molecules in the asymmetric unit are packed anti-parallel, with the three domains of each molecule covering a total surface area of 22,030 Å^2^. The M- and C-domain have an interface area of 567 Å^2^, with nine hydrogen bonds and one salt bridge. The interface area between the N- and M-domains (787 Å^2^) involves multiple interactions, including 14 hydrogen bonds and three salt bridges.

### Main structural features of the GG-SpaD domains

**Fig. 2:**
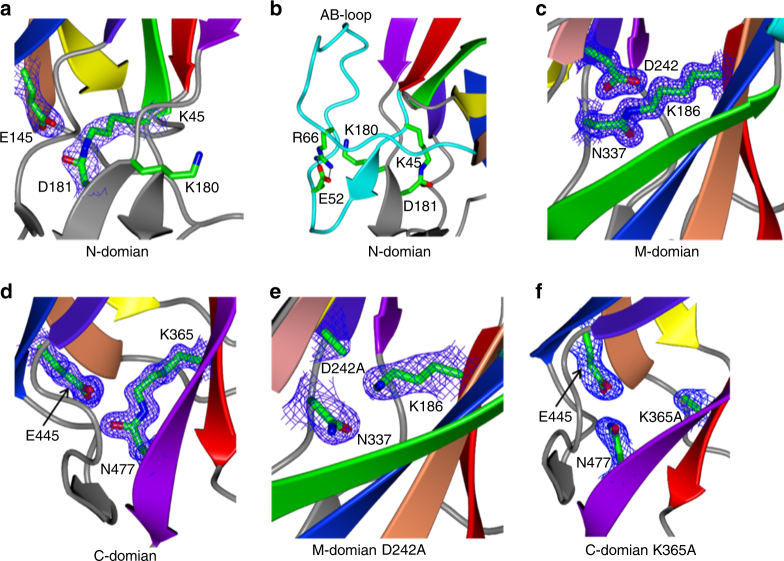
Internal isopeptide bonds in the WT and mutant GG-SpaD crystal structures. The electron density (2Fo-Fc) map is contoured at 1.5 σ. **a** An internal isopeptide bond in the N-domain is found between K45 and D181, with the catalytic Glu145 in close proximity. **b** The N-domain isopeptide bond is found adjacent to the linking lysine K180, which is covered by the AB loop. A salt bridge between E52 and R66 ties together the N- and C-termini of the AB loop. **c** In the M-domain, the two β-sheets of CnaA fold are connected by an internal isopeptide bond formed between K186 and N337 via the proximal catalytic D242. High-resolution (1.5 Å) data of chymotrypsin-digested GG-SpaD (GG-SpaD_MC_) are used to provide better representation. **d** In the C-domain, an internal isopeptide bond that connects the first and last β-strands of the CnaB fold is formed between K365 and N477, with the participation of the nearby catalytic E445. **e** Alanine substitution of catalytic Asp242 in the M-domain prevents isopeptide bond formation between K186 and N337. **f** Alanine substitution of Lys365 breaks the K365-N477 isopeptide bond in the C-domain with minimal distortion to the overall tertiary structure

In the structure of the N-domain there is clear continuous electron density between Lys45 and Asp181 within the C-terminal region of the first (A) and last (G) β-strands, which indicates the formation of an isopeptide bond in a *trans* configuration (Fig. 2a). Surrounding this isopeptide is a cluster of hydrophobic residues. Catalytic Glu145 from β-strand E is situated in close proximity and forms a hydrogen bond with the O moiety of the K–D isopeptide bond. Asp181 is found within the uncommon YPKD pilin motif. This motif also contains the lysine (Lys180) that is involved in intermolecular isopeptide bond formation during pilus assembly, a role we confirmed by amino-acid substitution (K180A) (Supplementary Fig. 1). The side chain of Lys180 points outward from the core of the CnaB fold into a cavity formed by the extended and hook-shaped AB loop (Fig. 2b). Residues Glu52 and Arg66 form a salt bridge between the two ends of the AB loop, and thus cover Lys180. Found diagonally opposite the isopeptide bond, on the other end of the N-domain, is the BC loop, which is formed by two short α-helices and interacts with the BC loop of the M-domain at the interface of the two domains (Fig. 1b). The FG loop is opposite the BC loop and directed away from the core fold, but also contains a short β-hairpin adjacent to the AB loop near the N-terminus.

For the M-domain, continuous electron density shows the formation of a *trans* isopeptide bond between Lys186 and Asn337, with a catalytic Asp242 lying nearby (Fig. 2c). A cluster of 12 hydrophobic residues surround this isopeptide bond. Functionally, the K–N isopeptide bond probably brings stability to the M-domain by covalently connecting the two β-sheets of the CnaA fold (Fig. 1d). Here, the β-sheets comprise four (A, B, E, and D) and five (D’, D”, C, F, and G) anti-parallel β-strands (Fig. 1b). The AB and EF loops in the C-terminal region of the M-domain interact with the C-domain at their interface. A pair of α-helices at the N-terminal end forms the DE loop that interacts with the BC loop of the N-domain. Two additional β-strands (D’ and D”) not part of the β-sandwich core of typical Ig-like domains are found between β-strands D and E.

In the C-domain structure, the observation of continuous electron density between Lys365 and Asn477 confirms that a *cis* isopeptide bond is present and located in the first (A) and last (G) β-strands of the CnaB fold (Fig. 2d). Catalytic Glu445 forms a hydrogen bond with the O moiety of the K–N isopeptide bond in a hydrophobic pocket. At the N-terminal of the C-domain, short loops (FG and DE) form an interface with the C-terminal region of the M-domain. Also found at this domain interface are two anti-parallel β-strands in the BC loop that align perpendicularly to the core fold of the C-domain (Fig. 1b). Conspicuously, residues (480–485) from part of the LPXTG sorting motif extend beyond the C-domain at the C-terminal end.

To study the effect of lysine and catalytic glutamate or aspartate on isopeptide bond formation and structural stability, alanine-substituted mutants were prepared (Supplementary Table 1). X-ray diffraction data were collected for two mutant proteins (D242A in the M-domain and K365A in the C-domain), and though no isopeptide bonds were present in each, their overall tertiary structures remain unchanged (Fig. 2e, f).

### Structural conformations of the N-domain

**Fig. 3:**
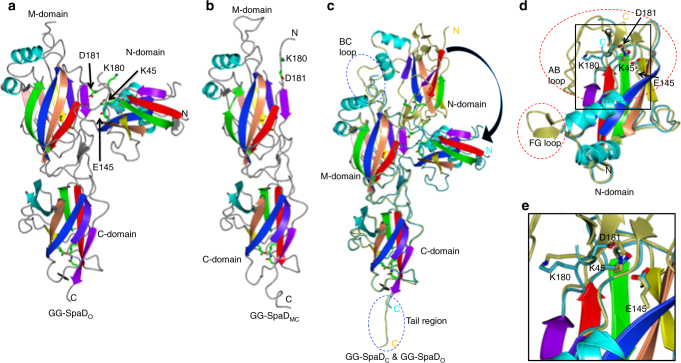
Open conformation structure of GG-SpaD with a repositioned N-domain. **a** The GG-SpaD crystal structure in the open (bent) conformation (GG-SpaD_O_) is shown in ribbon representation. Core β-strands of the N-, M-, and C-domains are in rainbow colors. Linking K180 and the residues for internal isopeptide bonds (shown in stick representation) are indicated by a black arrow. AB and FG loops appear disrupted in the N-domain, leaving K180 fully exposed and the internal K–D isopeptide bond unformed. Internal isopeptide bonds in the M- and C-domains are intact. **b** Crystal structure of the C-terminal GG-SpaD fragment obtained by limited proteolysis and containing the M- and C-domains (GG-SpaD_MC_). **c** Superimposition between the GG-SpaD_O_ and GG-SpaD_C_ crystal structures. The N-domain in GG-SpaD_O_ is swung out by about 100°. The M- and C-domains in GG-SpaD_O_ are intact, with only subtle structural deviations (encircled by a blue dashed line). These include the BC loop in the M-domain, which appears disordered and is no longer in contact with the N-domain, and the C-terminal tail with a partial sorting motif, which now lacks a discernible electron density. **d** Superimposition between the N-domains of GG-SpaD_C_ (gold) and SpaD_O_ (cyan). Regions with the most structural perturbations are encircled by a red dashed line. AB and FG loops in GG-SpaD_O_ are both disordered. **e** An enlarged view of the major changes around the residues forming an isopeptide bond. D181 has shifted away by about 5 Å and can no longer form an isopeptide bond with K45. K45 now hydrogen bonds with the side chain of catalytic E145 and the main chain of Q153. K180 is released from its hydrogen bonding that occurs with the AB loop and through the main chain of E52, which makes a salt bridge with R66

In the hexagonal crystal structure of GG-SpaD, the N-domain is angled about 100° from the M- and C-domains. This bent shape is labeled GG-SpaD_O_ for the open conformation (Fig. 3a), and here the N-domain no longer has an isopeptide bond. We also solved a GG-SpaD structure where only the M- and C-domains are present (GG-SpaD_MC_) (Fig. 3b). Found in common with the GG-SpaD_C_, GG-SpaD_O_, and GG-SpaD_MC_ structures is that the arrangement of the M- and C-domains is linear and nearly identical, yielding a RMSD of less than 1 Å (Fig. 3c). In GG-SpaD_C_, the N-domain is aligned linearly with the M- and C-domains (Fig. 1b), taking on the elongated rod-like shape commonly seen in other Gram-positive backbone pilins^21^. Contrastingly in GG-SpaD_O_, the N-domain is nearly perpendicular to the two other domains and situated midway along the M-domain (Fig. 3a, c). Due to this repositioning, the overall length of the GG-SpaD pilin is shortened to 90 Å and the total surface area is reduced to 19,679 Å^2^. However, the overall CnaB fold of the N-domain displays no difference between the closed and open conformations, as their RMSD is less than 1 Å (Fig. 3d, e). Remarkably, the bent orientation of the N-domain has never been observed before in the crystal structures of other backbone pilins.

Compared to the interface interactions between the closed N- and M-domains, the open N-domain has lost most of these contacts. Instead, it has established a new interface with the middle and C-terminal end of the M-domain (628 Å^2^) that involves nine hydrogen bonds and four salt bridges. Also, the BC and EF loops of the open N-domain interact with the AB loop and core fold of the M-domain. Some regions of the open N-domain structure appear disordered, e.g., the FG loop with the short β-hairpin and the AB loop with a salt bridge (Glu52-Arg66) covering the linking Lys180 that is now exposed to solvent (Fig. 3d, e). Because Asp181 has shifted about 5 Å away from Lys45, isopeptide bond formation is no longer possible between these two residues. Instead, Lys45 forms a hydrogen bond interaction with catalytic Glu145 (Fig. 3d, e). A major structural alteration in the N-domain involves the segment of residues Tyr178 to Leu183, which includes the YPKD pilin motif and links the M-domain. This linker in GG-SpaD_C_ forms a three-stranded β-sheet with the AB and EF loops at the interface with the M-domain. In GG-SpaD_O_ this short β-sheet has collapsed.

### Biophysical analysis of N-domain motion

**Fig. 4:**
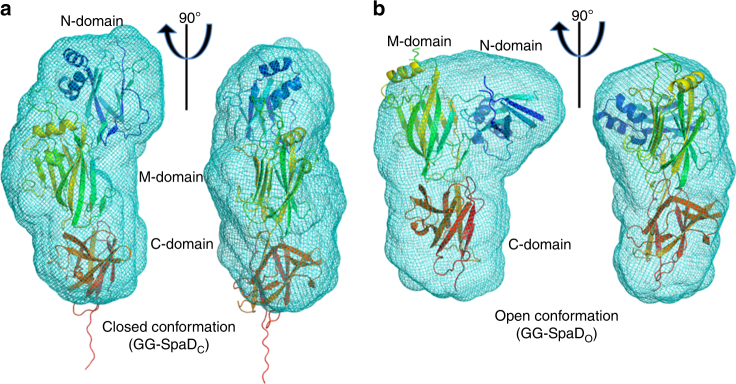
SAXS analysis of GG-SpaD. **a** Two orthogonal views of the overlap of the SAXS envelope (cyan) and the best-fitted crystal structure model (ribbon representation) for the closed conformational state of GG-SpaD (GG-SpaD_C_). **b** As in (**a**), but for the open conformational state of GG-SpaD (GG-SpaD_O_). The ribbon representation of the crystal structure model is in blend through colors, starting with blue at the N-terminal and ending with red at the C-terminal. The N-, M-, and C-domains are identified

To gauge whether the closed and open conformations of the N-domain exist in solution, we conducted small-angle X-ray scattering (SAXS) analysis of the GG-SpaD protein. Results from the scattering profile showed that GG-SpaD assumes an elongated shape with a radius of gyration (Rg) of 28 Å and a maximal particle dimension (D-max) of 9.23 nm (Supplementary Fig. 2, Supplementary Table 2), and thus corresponds to the closed conformation (Fig. 4a). In order to mimic the conditions used to obtain the open conformation crystal structure form, we carried out the SAXS experiment in the presence of added salt (1 M NaCl). As shown in Fig. 4b, GG-SpaD now resembles the open conformation (Rg of 25Å and D-max of 7.92 nm; Supplementary Fig. 2). SAXS models of the closed and open shapes of GG-SpaD superimpose well on those of the respective crystal structures (Supplementary Fig. 2, Supplementary Table 2). This suggests that the crystal structures of the two conformational states for GG-SpaD probably represent the native proteins in solution.

**Fig. 5:**
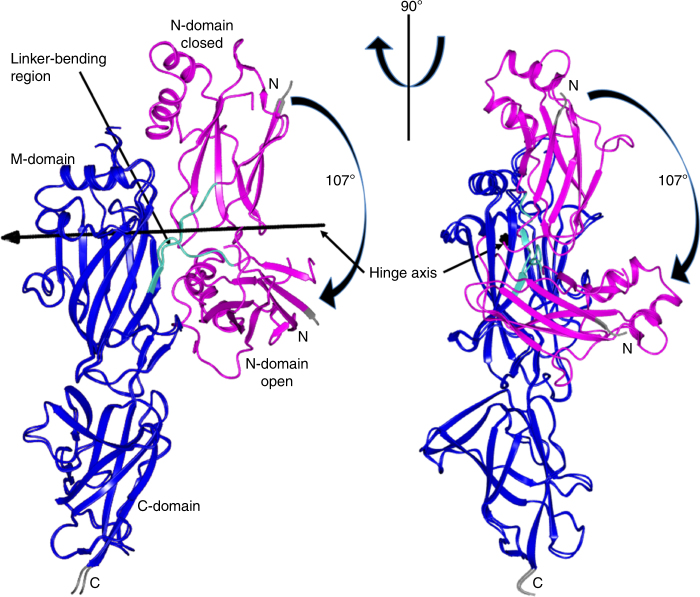
Two orthogonal views of GG-SpaD exemplifying N-domain motion. The predicted closed and open conformations of GG-SpaD obtained using the DynDom program are superimposed. The mobile N-domain (magenta) is connected to the static M- and C-domains (blue) through a hinge region (cyan). N- and C-termini are indicated by gray coloring. A black line with an arrow-head shows the location of the hinge axis. N-domain rotation (107°) is indicated by a black arrow

For an in silico glimpse into the possible movement of the N-domain, we used the DynDom program^49^ to analyze the closed and open conformation crystal structures of full-length GG-SpaD. Output from DynDom suggests a large 296-residue static domain comprising residues 184–479 with a backbone RMSD of 0.56 Å (i.e., M- and C-domains) and a smaller 145-residue mobile domain containing residues 39–183 with a RMSD of 1.53 Å (i.e., N-domain) (Supplementary Table 3). DynDom also predicted a hinge region for interdomain bending comprising residues 179 to 186, which we have identified as a linker segment between the N- and M-domains. As depicted in Fig. 5, the N-domain rotates 107° around the hinge axis passing between β-strands B and E of the M-domain adjacent to Leu183 of the linker. Further DynDom analysis indicates that N-domain movement follows a standard closure motion of 77%.

**Fig. 6:**
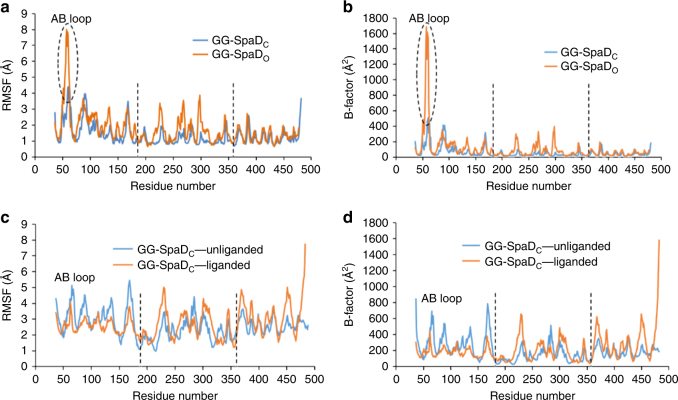
MD simulation analysis of the closed (GG-SpaD_C_) and open (GG-SpaD_O_) conformation structures of GG-SpaD. **a** RMS fluctuation of residues of the closed (blue) and open (orange) conformations of monomeric GG-SpaD during MD simulation. Boundaries for the N-, M-, and C-domains are identified by a vertical black dashed line. Regions with relatively high RMSF are observed within the N-domain of GG-SpaD_O_, with maximum fluctuation at the AB loop (encircled by a black dashed line). **b** B-factors for residues in the closed (blue) and open (orange) conformations of monomeric GG-SpaD. **c** RMSF analysis of dimeric GG-SpaD_C_. The dimeric form of GG-SpaD_C_ is represented by the molecular arrangement of the two molecules in the asymmetric unit of the orthorhombic crystal. The N-domain of one molecule has the C-terminal tail in the groove of the AB loop (ligand-docked; orange), whereas that of the other molecule does not (ligand-free; blue). **d** B-factors for residues of dimeric GG-SpaD. Labeling is as in (**c**)

To examine the domain flexibility of GG-SpaD, molecular dynamics (MD) simulations of models for the GG-SpaD_C_ and GG-SpaD_O_ crystal structures were performed (Fig. 6). Values from RMSF (root mean-square fluctuation) (Fig. 6a) and B-factor (Fig. 6b) calculations indicate that, in comparison with the M- and C-domains, there is extra conformational fluctuation in the N-domain. Also, the degree of structural flexibility for the N-domain is greater in GG-SpaD_O_ than in GG-SpaD_C_. Moreover, the AB loop in the N-domain that showed maximum flexibility now appears to be stabilized upon becoming liganded to the C-terminal tail of an adjacent subunit, such as seen in the crystal packing (Fig. 6c, d).

### Structural implications for SpaFED pilus assembly

**Fig. 7:**
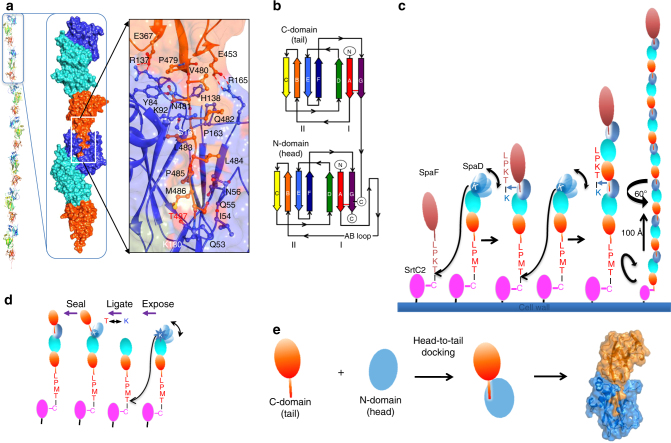
Structural model for the backbone assembly of GG-SpaD into the SpaFED pilus. **a** Ribbon representation of the GG-SpaD subunits in the SpaFED pilus structure is based on the orientation of the two asymmetric unit molecules (outlined by rectangle) (left panel). A surface representation of two adjoined GG-SpaD subunits (N-domain in blue, M-domain in cyan, and C-domain in orange) and an accompanying image of their interface interactions are shown enlarged (middle and right panel). Modeling M486 and T487 into the GG-SpaD structure reveals that a covalent bond occurs between K180 and T487 (right panel). **b** Topology diagram highlighting the interaction between the C-terminal tail in the C-domain of one GG-SpaD subunit and the groove of the AB loop in the N-domain of another adjoining GG-SpaD subunit. The core β-strands of two β-sheets are labeled A to G using rainbow colors. **c** Schematic representation of GG-SpaD incorporation into the SpaFED pilus backbone structure. The N-, M-, and C-domains of GG-SpaD are indicated by color (blue, cyan, and orange, respectively). The pilus-specific SrtC2 sortase (magenta) and the GG-SpaF tip pilin (pink) are also indicated. SrtC2 cleaves the bond between the threonine (T) and glycine (G) residues of the LPMTG motif peptide in GG-SpaD and then catalyzes the formation of an intermolecular K–T isopeptide bond via the side chain of the linking lysine (K180) in another incoming GG-SpaD subunit. **d** Depicted is how both N-domain movement and the unstructured AB loop expose K180 for nucleophilic attack during isopeptide bond formation (sortase-mediated ligation). The C-terminal tail is docked into the groove of the N-domain, whereupon the AB loop then regains a structured form and seals together the adjoining GG-SpaD subunits, the final stage of the expose-ligate-seal mechanism. The molecular arrangement in the crystal lattice indicates that each GG-SpaD subunit in the assembled SpaFED pilus has a projected 60° rotation and 100 Å translation. **e** Blind docking of the N-domain (head) with the C-domain (tail) generates the same head-to-tail configuration as seen within the crystal lattice. A representative model of the docked N- and C-domains corresponds to one of the ten best ClusPro predictions

The two GG-SpaD molecules in the asymmetric unit for the closed conformational state are stacked linearly head-to-tail, with the C-domain (tail) of one GG-SpaD subunit docked into a groove-like hydrophobic opening in the N-domain (head) of the neighboring subunit (Fig. 7a). Also located in the groove of the N-domain is the linking lysine (Lys180), which lies within covalent bonding distance of the C-terminal LPXTG-motif threonine (Thr487) in another pilin subunit (Fig. 7a, b). The interface between the C- and N-terminal regions of adjacent GG-SpaD subunits encompasses a surface area of ~765 Å^2^, with eight hydrogen bonds and two salt bridges (Supplementary Table 4). Three sides of the cavity for docking the C-terminal tail are built from the last two β-strands along the side of the β-sandwich between the FG and EF loops, and the lengthy AB and BC loops. The crystal packing of the open conformation revealed that the single GG-SpaD molecule in the asymmetric unit lacks a linear pilus-like structure. Noticeably, conformational changes involving disorder exist in the AB, FG, and BC loops when the C-terminal tail of a symmetry-related molecule is unavailable for docking (Fig. 3).

The angle between the N-domain and C-domain of neighboring pilin subunits is 126°, and thus it is rather similar to the N- and M-domain (150°) and M- and C-domain (120°) angles for a single subunit. For the assembled SpaFED pilus structure, the adjacent subunits are each translated 120 Å and rotated ~60° along the axis, with a diameter of 30–50 Å (Fig. 7a, c). Presumably during the pilus assembly process, the SpaFED C-type sortase (SrtC2) cleaves the bond between Thr487 and Gly488 of the LPMTG-motif peptide in GG-SpaD and then catalyzes intermolecular isopeptide bond formation between Thr487 and the side chain of the linking lysine (Lys180) from the adjacent pilin (Fig. 7c). In this instance, it seems that the closure motion observed for the N-domain in the open conformational state is necessary for assembling GG-SpaD pilin subunits into a pilus fiber. Here, we propose that the process of sortase-mediated pilus polymerization follows an expose-ligate-seal mechanism (for details, see Fig. 7d and Discussion).

Crystal structure and MD simulation analysis of GG-SpaD suggests that the docking of the C-terminal tail from the adjacent subunit has a stabilizing effect on those regions of N-domain involved with intermolecular interactions, which itself is primarily through an interaction between the AB loop and C-terminal tail. To assess this possibility, we docked the N-domain (head) and C-domain (tail) of GG-SpaD_C_ (i.e., blind docking) by using the ClusPro protein–protein docking tool^50^. Among the top ten best low-energy conformational models that were predicted, one shared a strong resemblance to the GG-SpaD_C_ crystal structure (Fig. 7e).

### Structural comparison of GG-SpaD

**Fig. 8:**
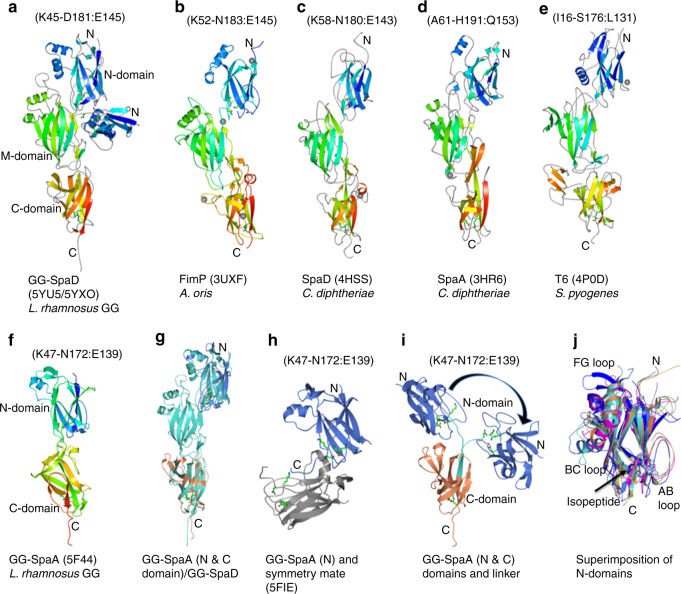
Structural comparison of GG-SpaD_C_ and GG-SpaD_O_ with related three-domain backbone pilins and two-domain GG-SpaA. Ribbon representation of crystal structures of the N-, M-, and C-domains is in blend through colors, with blue at the N-terminal (N) and red at the C-terminal (C). Metal location is indicated by a gray sphere. Residues involved with isopeptide bonding (or at equivalent positions) are shown in sticks (residue numbering is shown above the respective structures). **a–e** Three-domain backbone pilins (GG-SpaD_C_ and GG-SpaD_O_, *A. oris* FimP, *C. diphtheriae* SpaD and SpaA, and *S. pyogenes* T6) that contain the N-domain in their crystal structure. **f** Two-domain backbone pilin GG-SpaA from *L. rhamnosus* GG. **g** Superimposition of GG-SpaA N- and C-domains (light blue and coral, respectively) with that of GG-SpaD (cyan). The superimposition results in rmsd values of 2.2 Å (N-domain) and 1.8 Å (C-domain). **h** GG-SpaA N-domain (light blue) with a bent linker docked into the AB loop-formed groove of its adjacent symmetry-mate molecule (gray). **i** Two possible conformational states of the GG-SpaA N-domain generated by the superimposition of the crystal structures of full-length GG-SpaA (**f**) and truncated GG-SpaA N-domain (**h**), and guided by part of the linker region (cyan) of the C-domain. The GG-SpaA N-domain appears to rotate 173° based on a DynDom analysis for domain motion. **j** Superimposition of the crystal structures of N-domains from three-domain backbone pilins, i.e., GG-SpaD (magenta), *A. oris* FimP (coral), *C. diphtheriae* SpaD (gold), *C. diphtheriae* SpaA (cyan), *S. pyogenes* T6 (light blue), and two-domain GG-SpaA (blue). The location of the internal isopeptide bond in the CnaB domain is conserved, but with some deviations in the AB, BC, and FG loops

As determined by a DALI^51^ search of the PDB, a number of three-domain pilins are related to the structure of GG-SpaD (open/closed conformations and individual domains) (Supplementary Table 5), although the amino-acid sequence identity was low (in the range of 14–27%). In general, the CnaB fold of the N- and C-domains and the CnaA fold of the M-domain are well conserved across the three-domain pilins^21^, which includes GG-SpaD even with its overall structural orientation showing some differences (Fig. 8). The top three hits were *Actinomyces oris* FimP^37^ and *C. diphtheriae* SpaD^33^ and SpaA^32^ (Supplementary Table 5). Three-domain *Streptococcus pyogenes* T6 backbone pilin^40^ was eighth on the list. When we performed DALI searches with individual domains, the best fits for the N-, M-, and C-domains were for those from *C. diphtheriae* SpaD^33^, *Streptococcus agalactiae* BP-2a^35^, and two-domain GG-SpaA^29^, respectively (Supplementary Table 5). However, we never obtained a match for the N-domain when DALI searches were done using the full-length GG-SpaD_O_ structure. Although GG-SpaD_C_ (Fig. 8a) is topologically similar to other three-domain pilins (Fig. 8b–e), certain differences exist (see further in Supplementary Note 1).

**Fig. 9:**
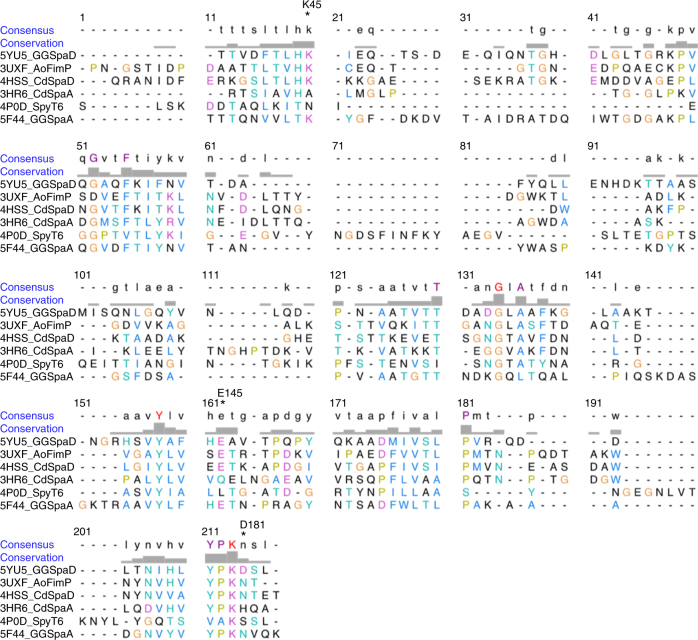
Multiple sequence alignment showing the key residues for an internal isopeptide bond in the N-domain of GG-SpaD and its structural homologs. Amino-acid sequences for N-domains from the crystal structures of three-domain backbone pilins (i.e., GG-SpaD, *A. oris* FimP, *C. diphtheriae* SpaD and SpaA, and *S. pyogenes* T6) and two-domain GG-SpaA are aligned using the MUSTANG (MUltiple STructural AligNment AlGorithm) program. Residues involved with isopeptide bonding are marked by an asterisk (*) and denoted according to the residue number of GG-SpaD. Residues for internal isopeptide bond formation are absent in the *C. diphtheriae* SpaA and *S. pyogenes* T6 pilins

Like GG-SpaD, two-domain GG-SpaA also has an intact isopeptide bond in the CnaB fold of its N-domain (Fig. 8f, g), but which is unformed when the connecting linker region bends in the absence of the C-domain^29^ (Fig. 8h). Interestingly, a bent type of conformation for the N-domain of GG-SpaA (Fig. 8i) could be established by aligning the C-terminal part of the linker region in the truncated form (Fig. 8h) with that of the C-domain in the full-length structure (Fig. 8f). For this, a DynDom analysis of domain motion shows a rotation of about 173° between the two domains, with bending at residues 171–181 and a closure motion of 74% (Fig. 8i). As seen by the superimposed structures (Fig. 8j) of N-domains from various backbone pilins (Fig. 8a–f), the locations of the isopeptide bonds are well conserved in these CnaB-type folds, but with obvious deviations in the loop regions. Clearly, this structural comparison indicates that the K–D isopeptide bond in the N-domain is only associated with the GG-SpaD pilin, an observation further substantiated by the structure-based sequence alignment of N-domains (Fig. 9).

Incidentally, although the backbone pilins of the Gram-negative chaperone-usher^52^ and type V^53^ pili consist of Ig-like folds, a DALI search did not reveal any structural similarity with GG-SpaD as their overall fold topologies differ from one another. Moreover, since the backbone pilins of the Gram-positive type IV pili contain a structurally dissimilar scaffold with a distinct β-sheet topology (αβ fold)^54^, these were not among the DALI hits.

### AB loop disorder in the head-to-tail docking of GG-SpaD

**Fig. 10:**
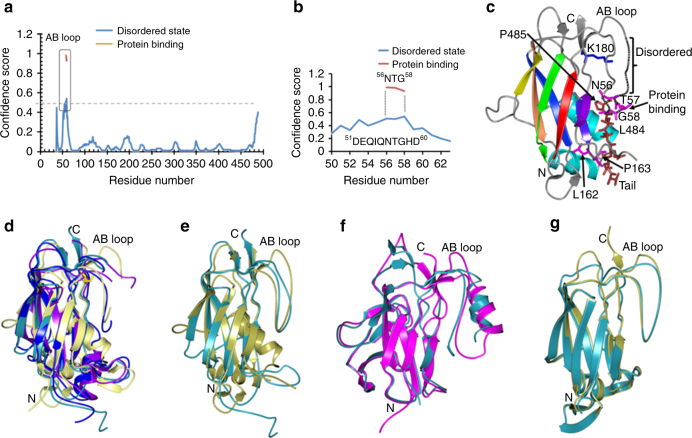
In silico analysis of the AB loop in the N-domain. **a** Intrinsically disordered regions in GG-SpaD were predicted with the DISOPRED program. Regions in the GG-SpaD sequence reflecting disorder (blue) and protein binding (orange) are shown in the disorder profile. A horizontal gray dashed line marks the level of confidence (0.5). **b** An enlarged view of predictions for disorder in the AB loop (^51^DEQIQNTGHD^60^) and for its protein-binding site (^56^NTG^58^). **c** The predicted locations of disorder (black dotted line) and protein binding (magenta sticks) within the GG-SpaD N-domain are indicated. Regions of contact between the C-terminal tail residues (488–485) of the C-domain (brown sticks) and the protein-binding residues (NTG) are shown. A conserved region (L484-P485) within the FG loop is labeled (sticks). **d** Superimposition of known N-domain structures from three-domain pilins. The AB loop in the N-domains of GG-SpaD (5YU5_B; gold), *C. diphtheriae* SpaD (4HSS_B; cyan), *A. oris* FimP (3UXF; blue), and *C. diphtheriae* SpaA (3HR6; purple) appears disordered when the C-terminal tail is missing or improperly docked in the crystal unit. **e** Superimposition of N-domain structures from GG-SpaD (5YU5_A; gold) and *C. diphtheriae* SpaD (4HSS_A; cyan) show that the AB loop is structured in the presence of a properly inserted C-terminal tail. **f** Superimposition of N-domain structures from the four-domain *Streptococcus pneumoniae* RrgB pilin in the presence (2Y1V; magenta) and absence (3RPK; dark cyan) of a C-terminal tail. **g** Superimposition of N-domain structures from the two-domain GG-SpaA pilin show an ordered AB loop in the presence of a partial C-terminal tail (5F44; gold) or a domain linker that mimics a C-terminal tail (5FIE; cyan)

As the disorder in the AB loop appears important for assembling the GG-SpaD subunits, we analyzed this further through in silico predictions. Disordered regions were predicted for the AB loop using the DISOPRED program^55^, with residues ^51^DEQIQNTGHD^60^ being identified. A short segment (^56^NTG^58^) within this disordered region was further identified as a protein-binding site in the AB loop (Fig. 10a, b). This intrinsically disordered segment in the AB loop apparently represents a molecular recognition feature (MoRF), as it undergoes a disorder-to-order transition and becomes structured upon binding to its partner protein, i.e., the C-terminal tail of GG-SpaD. BLAST searches of the UniProt Reference Clusters database at the UniprotKB site (www.uniprot.org) using GG-SpaD sequence found that the NTG segment is an invariant feature among other sortase-dependent pilins containing the YPKN pilin motif (Supplementary Fig. 3). As seen from both the docked GG-SpaD dimers in the crystal unit cell and the predicted docking model (Fig. 7), the NTG peptide is located at the middle of the AB loop (Fig. 10c) in the groove where the C-terminal tail would dock and form an interaction with the LPMT peptide. Additional protein interface analysis of GG-SpaD by PISA^56^ revealed that Asn56 of the NTG peptide would have the greatest stabilizing effect (Supplementary Table 4). Also found important is a conserved segment (^162^LP^163^) within the FG loop (Supplementary Fig. 3) that forms an L-shaped bend, which apparently makes partial contact with the NTG and LPMT peptide regions (Fig. 10c). Analysis of other related three-domain backbone pilins and two-domain GG-SpaA also show maximum disorder in the AB loop region (Supplementary Fig. 4).

We further compared the AB loop in the N-domains by structural superimposition (Figs. 8j, 10d–g). Unlike the other lengthier loops in pilin structures, the AB loop of the N-domain often lacks the secondary structure that would let it act as gate, and thus exposing the linking lysine during isopeptide bond formation. Nonetheless, it appears that both ends of the AB loop are tied together, either by a salt bridge as in GG-SpaD (Fig. 2b) or by a disulfide bond as in corynebacterial SpaA^32^ (Fig. 10d). Further, the linking lysine in the AB loop-formed groove seems to be engaged in a hydrogen bond with the main chain of the tyrosine residue in the YPKN pilin motif or with the AB loop just prior to intermolecular isopeptide bond formation. Interestingly, as with GG-SpaD, the AB loop in known pilin structures usually appears disordered when a structured C-terminal tail is not directed inwardly towards the groove region^29,31–33,36,37^. This might help explain why the N-domain is normally lost or removed in some backbone pilin crystal structures, as the residues for the C-terminal tail were not part of the recombinant protein.

## Discussion

The tertiary organization of GG-SpaD is much like other three-domain backbone pilins in Gram-positive bacteria^21^. However, apart from having some topological variances in loop lengths and positions, more notable structural differences in GG-SpaD lie with its N-domain. For instance, the N-domain has an intact isopeptide bond, which itself is atypically formed between lysine and aspartate and unlike the more common lysine-asparagine interaction. *C. diphtheriae* SpaD is the only other known three-domain backbone subunit whose N-domain also contains an intact isopeptide bond, albeit slow-forming and involving a K–N interaction^33^. K–D isopeptide bond formation in the N-domain of GG-SpaD likely also occurs slowly. The N-domain in two-domain backbone pilins^27,29,41^ is less flexible than in three-domain pilins, yet it also exhibits a K–N isopeptide bond. Among four-domain pilins, the N-domain isopeptide bond forms only during pilus assembly^28^, or when part of the C-terminal LPXTG motif is present and a pilus-like structure occurs in the crystal unit^31^.

Most backbone pilins contain the YPKN pilin motif peptide in their N-domain, wherein during pilus polymerization the lysine and asparagine residues participate in intermolecular and intramolecular isopeptide bond formation, respectively^21^. In GG-SpaD, an uncommon YPKD pilin motif exists in the N-domain and is conserved highly among other *Lactobacillus* strains. From the arrangement of GG-SpaD subunits as a native pilus backbone in the crystal unit and based on immunoblotting of residue-substituted protein (Supplementary Fig. 1), Lys180 of the YPKD peptide forms an intermolecular covalent link with Thr487 of the C-terminal LPXTG motif in an adjoining pilin. Moreover, continuous electron density supports that Asp181 (YPKD) forms an internal isopeptide bond with Lys45. Similar K–D isopeptide bonding has been observed in the C-domain of other backbone pilins, though not in their more flexible N-domain^29,37^. Presumably, other amino acids surrounding an internal isopeptide bond should impact whether an asparagine or aspartate residue is to be encoded genetically. Thus, the universal preference for a covalent interaction involving K–D over K–N by gut-adapted bacteria like *L. rhamnosus* GG might have evolved from the acidic conditions of the digestive tract.

Among the backbone pilins with a triple- or quadruple-domain structure, the N-domain is usually seen as the most flexible in nature, and thus, in many instances, its removal from the rest of the protein is needed for obtaining good X-ray-diffracting crystals^28,30,33–35,38,39^. Then again, for backbone pilins whose full-length structure has been solved^32,33,37^, the N-domain either lacks or slowly forms an isopeptide bond. Moreover, these full-length structures have revealed that the N-domain is somewhat laterally displaced from the long axis of the other domains, which themselves exhibit strong interface interactions with one another and show only a limited amount of motion. However, whereas this seems the case for three-domain backbone pilins like GG-SpaD, those with a two-domain organization are far more rigid in their structural makeup^27,29,41^. Nonetheless, while on its own or unliganded to the C-terminal tail of an adjoining pilin, the N-domain tends to be unstable in solution primarily due to the flexibility of the AB loop and the apparent absence of an isopeptide bond. Generally, this explains why the N-domain degrades while undergoing crystallization or does not form crystals at all. In this context, it was rather serendipitous that the N-domain structure of GG-SpaA was itself solved, as it appeared that in the crystal unit the C-terminal tail from the symmetry mate was a stabilizing influence on the mobile AB loop^29^. As for GG-SpaD in our present study, the solution of its full-length structure in the closed conformation was facilitated somewhat similarly.

Structurally, the GG-SpaA backbone pilin contains two CnaB domains, where K–N and K–D internal isopeptide bonds are present in the N- and C-domains, respectively^29^. By comparison, the overall structures of GG-SpaD and GG-SpaA bear little resemblance to each other, aside from having the same CnaB fold type for their N- and C-domains, though still lacking strong amino-acid sequence identity and exhibiting opposing kinds of intramolecular isopeptide bonds (K–N or K–D). More pointedly, the additional CnaA domain between the N- and C-domains in GG-SpaD makes this pilin larger than GG-SpaA, indicating that the overall structural topology between the SpaFED and SpaCBA pilus backbones is architecturally distinct.

Perhaps the most interesting aspect of this study was solving the GG-SpaD structure in the open conformational state, whereby the N-domain is no longer aligned with the M- and C-domains in a linear manner (closed conformation), but rather instead is positioned perpendicularly next to the middle region of the M-domain. Since this offers a unique structural perspective to backbone pilins, we suggest that the open conformation represents an intermediary snapshot of the backbone pilin during its sortase-mediated assembly into the SpaFED pilus (Fig. 7c, d). This viewpoint is well supported by the novel structural features of the bent N-domain: skewing away from the pilus axis; lacking the K–D isopeptide bond; exhibiting disorder in the AB loop that covers the linking Lys180; exposing Lys180 to the thioacyl sortase-pilin intermediate for accessibility to nucleophilic attack; displaying greater flexibility when an adjacent C-terminal tail is not docked in place; and causing the molecular arrangement of a pilus-like fiber in the crystal unit to be lost.

Moreover, we note that the inclusion of ionic NaCl during GG-SpaD crystal formation and SAXS analysis seems to have disrupted the normal interface interactions between the N- and M-domains, thereby favoring a repositioned N-domain. Along with our biophysical analysis for domain motion, such results suggest a flexible state for GG-SpaD, and this might be of mechanistic importance for the intermolecular pilin cross-linking during pilus polymerization. Here it seems that the flexible N-domain, and in particular its AB loop, functions as a gatekeeper to control access to the linking lysine by the C-terminal LPMTG-motif threonine. Our residue identification of a MoRF in the disordered region of AB loop further supports a functional role for the N-domain, which, similarly, is a structural aspect of the *S. agalactiae* GBS52 basal pilin^57^.

Our findings regarding the structural properties observed for the closed and open conformations of GG-SpaD have led us to posit a mechanistic model that outlines the early stages leading up to the assembly of backbone pilins into the SpaFED pilus structure (Fig. 7d). Here, the open conformation represents the active and unliganded form of GG-SpaD. While in this state the N-domain is structurally relaxed until which time it associates with the C-domain of another pilin. However, during the open state, the mobile AB loop of the N-domain is flexible, leaving the side chain of the linking lysine (Lys180) unprotected for favoring nucleophilic attack. This stage is called expose. The ligate stage follows when the proximal sortase-acyl intermediate lets a new amide bond form between the C-terminal LPXT-motif threonine (Thr487) of one pilin and the N-terminal pilin-motif lysine (Lys180) of another adjacent pilin. At this point, the N-domain takes on the closed conformation, whereupon the AB loop undergoes a conformational change and becomes ordered by engaging in stabilizing interactions with topologies from the adjoining C-domain, and through this it encloses the groove-like hydrophobic pocket of the N-domain. This final stage is called seal. Functionally, the ordered AB loop helps shelter the connecting K–T isopeptide bond that forms between the two backbone pilin subunits, which together now assume a linear and rod-like shape and begins to resemble a polymerized pilus structure (Fig. 7a–c). Since the absence of an internal isopeptide bond from the D181A substitution lacked any impact on pilus polymerization (Supplementary Fig. 1), this suggests that internal K–D isopeptide bond formation occurs when the N-domain takes on the linear conformation after the intermolecular K–T isopeptide bond is produced. Presumably, the internal isopeptide bond after forming will help secure in place the N-domain and minimize its flexibility during the sealing stage, much like what a supporting guy-wire does for a utility pole.

Finally, based on our interpretation of the closed and open conformation structures of *L. rhamnosus* GG-SpaD, we conclude that the expose-ligate-seal mechanism defines the process by which an incoming backbone pilin is covalently docked during sortase-dependent pilus assembly and one that is made possible by the coordinated movement of the N-domain and its functional AB loop. We further suggest that this three-stage model of pilin assembly might generally hold true for the sortase-dependent pili of other Gram-positive bacteria.

## Methods

### Protein preparation

Unprocessed *L. rhamnosus* GG (ATCC 53103) GG-SpaD protein is comprised of 517 amino acids (UniprotKB ID: A0A179XFF5). Recombinant full-length GG GG-SpaD (residues 36–485) was cloned and produced in *E. coli* BL21 (DE3) pLysS as described earlier^46,58^. For this, a putative 34-amino-acid signal peptide at the N-terminus and a 34-amino-acid C-terminal LPXTG-motif sorting signal (Fig. 1a) were replaced by cloning vector residues (MGRDPNS) and a hexahistidine tag (LPLEHHHHHH), respectively. Briefly, GG-SpaD-expressing cells were first disrupted by sonication and afterward the cell-free lysate was loaded onto a Ni^2+^-charged Hi-Trap chelating column (GE Healthcare) pre-equilibrated with buffer A (50 mM NaH_2_PO_4_, pH 7.4 and 150 mM NaCl). GG-SpaD protein was eluted by a linear gradient of buffer B (buffer A containing 400 mM imidazole), which was followed by size-exclusion chromatography using a Sephacryl S-200 (26/60) column (GE Healthcare) in buffer C (20 mM HEPES, pH 7.0, 150 mM NaCl, and 1 mM EDTA). A stable 33 kDa protein fragment of GG-SpaD was also generated by limited proteolysis using α-chymotrypsin (Sigma-Aldrich) as done previously^46^. N-terminal sequencing and mass spectrometric analysis confirmed that this 33 kDa protein encompasses residues 177–485, which corresponds to the M- and C-domains of GG-SpaD (GG-SpaD_MC_). Selenium derivatization of full-length GG-SpaD protein using a selenomethionine (SeMet) kit (Shanghai Medicilon Inc., China) was carried out according to the instructions of the manufacturer. GG-SpaD_SeMet_ protein was purified similarly as described above. Mutant proteins of GG-SpaD were made with the primers listed in Supplementary Table 1 using a site-directed mutagenesis kit (Agilent Technologies). Here, selected residues involved in the formation of isopeptide bonds were replaced with alanine. PCR amplification was carried out using Pfu Turbo DNA polymerase (Stratagene). Amplified DNA was first treated with DpnI at 37 °C to digest the template strand, and then transformed into the *E. coli* DH5α strain. Transformed plasmids with the desired nucleotide changes were confirmed by DNA sequencing. Mutant constructs were cloned into pET-28 + and expressed in *E. coli* BL21 DE3 cells. Purification of mutant GG-SpaD was done with essentially the same protocol as used for the wild-type (WT) protein.

### Crystallization, data collection, and processing

Initial tests for crystallizing GG-SpaD proteins were carried out with the Mosquito Crystal automated liquid-dispensing system (TTP Labtech) using commercially available crystal screening kits. Conditions showing crystals were subsequently optimized manually using the hanging drop-vapor diffusion method. For this, 1 µl aliquots of the protein and reservoir solutions were each mixed and equilibrated against 1 ml reservoir solution at 295 K. Further details about protein concentrations, buffers, and reservoir solutions are provided in Supplementary Table 6. GG-SpaD_MC_ protein generated from the limited proteolysis approach yielded orthorhombic crystals that diffracted to 2.0 Å resolution at the home X-ray source using a GeniX3D Cu HF (high-flux) microbeam X-ray generator set at 0.6 mA and 50 kV (Xenocs, France) and a MAR 345 image-plate detector (MAR Research, Germany)^46^. X-ray diffraction of the GG-SpaD_MC_ crystal form at the synchrotron source (ESRF BM14 beamline, Grenoble, France) gave 1.5 Å resolution (Table 1). For single-wavelength anomalous diffraction (SAD) data collection, sodium iodide-derivatized crystals of GG-SpaD_MC_ were generated as done before^46^. Orthorhombic and SeMet crystals of full-length GG-SpaD were obtained with a similar protocol along with further optimization. Full-length GG-SpaD was also crystallized in a hexagonal form by including 1 M NaCl as an additive in the protein buffer. High-resolution native and selenium multiwavelength anomalous dispersion (MAD) datasets of these crystals were collected at the synchrotron source, all of which diffracted anisotropically in the range of 2.3 to 2.9 Å resolution. Cryoprotectants used for the data collections are listed in Supplementary Table 6. X-ray diffraction data were indexed and integrated by using XDS^59^ and scaled with Aimless^60^ from the CCP4 program suite^61^. An anisotropic correction was performed for all the data (except GG-SpaD_MC_) using the STARANISO Server (http://staraniso.globalphasing.org), and then used in final cycles of model building and refinement with COOT^62^ and REFMAC5^63^, respectively. Optimal wavelengths for data collection at the synchrotron source (European Synchrotron Radiation Facility) were 0.95372 Å at the BM14 beamline (GG-SpaD_C_, GG-SpaD_O_, GG-SpaD_MC_, and GG-SpaD_K365A_) and 0.97625 Å (GG-SpaD_D242A_) at the ID30B beamline. Ramachandran statistics for the refined crystal structures showed 96–98% and 1–3% of residues in favored and allowed regions, respectively, with no outliers (0%) detected (Supplementary Table 7).

### Structure determination

Initially, full-length GG-SpaD was crystallizable, but X-ray diffraction of the protein crystals at the home source led to an anisotropic pattern of streaky spots that was not indexable. The use of limited proteolysis had produced a stable 33 kDa protein fragment (GG-SpaD_MC_), whose crystals diffracted up to 2 Å and 1.5 Å resolution using home^46^ and synchrotron sources, respectively (Table 1). GG-SpaD_MC_ crystals belonged to the primitive orthorhombic space group (*P*2_1_2_1_2_1_) with two molecules in the asymmetric unit. Structure solution for GG-SpaD_MC_ was via iodide-SAD phasing with the diffraction data obtained at the home source^46^. Model refinement of GG-SpaD_MC_ was performed with the high-resolution native data collected at the synchrotron source (Table 1). Eventually, with the combined use of optimal crystallization conditions and data collection parameters, processable data for full-length GG-SpaD were generated. These crystals diffracted anisotropically up to 2.3 Å resolution at the synchrotron source and belonged to the same primitive orthorhombic space group as above, and as well contained just two molecules in the asymmetric unit. Structure solution by molecular replacement (MR) using PHASER^64^ with GG-SpaD_MC_ as a search model yielded insufficient density for the N-domain. Instead, data collected from the GG-SpaD_SeMet_ crystal at the peak, remote, and inflection wavelengths were used for MAD phasing with AUTOSHARP^65^ (Supplementary Table 8), which is integrated in CCP4^61^ and allowed the core β-strands of the N-domain to be built. With this now as a partial model for MR-SAD phasing, data collected at the peak wavelength of selenium were used to build the side chains and loops of the N-domain structure. All the residues were clearly seen in the electron density map for the two GG-SpaD molecules in the orthorhombic crystal unit, though molecule A displayed a relatively higher B-factor value than molecule B. The FG loop in the N-domain and two residues at C-terminus end were disordered in molecule A, whereas molecule B had only a few disordered residues in the AB loop of the N-domain. This solved crystal structure of full-length GG-SpaD is denoted as GG-SpaD_C_ for a closed conformation. The model of full-length GG-SpaD was refined against the anisotropically truncated native data (Table 1).

During the process of improving the diffraction quality of full-length GG-SpaD crystals, a hexagonal crystal form was identified when additional salt (1 M NaCl) was included with the protein-buffer solution in the crystallization screening experiments. These crystals diffracted to 2.5 Å, but belonged to the primitive hexagonal space group (*P*6_5_22) and exhibited only one molecule in the asymmetric unit. However, structure solution by MR using GG-SpaD_MC_ as a search model now gave a traceable difference electron density map, though for a differently placed N-domain that is situated perpendicular to the positioning of the M- and C-domains. The N-domain model from the orthorhombic form of GG-SpaD was also used as a reference structure for model building. After final rounds of model building and refinement using the anisotropically truncated data for the single molecule of GG-SpaD in the asymmetric unit, disorderedness was observed in the AB (residues 46–67) and FG (residues 164–171) loops of the N-domain, EF loop (residues 226–233) of the M-domain, and three residues at the C-terminal end (residues 482–485). This full-length GG-SpaD crystal structure is denoted as GG-SpaD_O_ for an open conformation. The model of full-length GG-SpaD (orthorhombic) was used in the MR and refinement of the mutant GG-SpaD structures (Table 1).

### Small-angle X-ray scattering (SAXS) analysis

SAXS analysis was carried out to assess whether the GG-SpaD_C_ and GG-SpaD_O_ crystal structures are representative of proteins in solution. For this, purified recombinant GG-SpaD protein (8.5 mg mL^-1^ and 17 mg mL^-1^) was dissolved in a buffer containing 20 mM HEPES (pH 7) and 150 mM NaCl. To simulate the conditions for obtaining the GG-SpaD_O_ structure, a sample of fresh GG-SpaD protein was also incubated with 1 M NaCl in the same buffer above for 24 h prior to the SAXS analysis. Scattering data were generated with a SAXS Space small-angle X-ray scattering instrument (Anton Paar, Austria) equipped with a sealed tube (line collimation) X-ray source and a one-dimensional CMOS Mythen detector (Dectris, Switzerland) at the SAXS facility in the Institute of Microbial Technology (IMTECH), Chandigarh, India. Data for each sample were obtained by a 20-min exposure (two 10-min frames) at 20 °C. Calibration and correction of the scattering patterns for the primary beam position was done with SAXStreat software (Anton Paar). A buffer scan was recorded before the sample runs under the same conditions and subtracted from the collected data using SAXSquant software (Anton Paar). Estimates of the radius of gyration (Rg) and maximal particle dimension (D-max) were obtained using programs (PRIMUS and GNOM, respectively) within the ATSAS software package. The program DAMMIF was used to generate low-resolution ab initio envelopes using the pair distribution function *P*(r), which were further averaged with the program DAMAVER. The normalized spatial discrepancy (NSD) value (computed using SUPCOMB) was 0.6 as an average of ten bead models (Supplementary Table 2).

### Molecular simulations and domain motion analysis

Molecular dynamics simulations of domain flexibility in GG-SpaD were performed using the AMBER14 program package (http://ambermd.org/)^66^, with AMBER14 force field and TIP3 potential for analyzing proteins and water molecules, respectively. Full-length models of the GG-SpaD_C_ and GG-SpaD_O_ crystal structures were hydrated in a 10 Å cubic water box, with the net charge neutralized by including an appropriate number of Na^+^ ions. After incremental heating to 310 K, simulation runs lasted 100 ns, with a time step of 10 fs and using the NPT ensemble at 310 K and 1 bar. A Nosé-Hoover thermostat (coupling constant *t*_t_ = 2.524, 25) and a Parrinello-Rahman barostat (*t*_p_ = 5.0 ps) were used to control the temperature and pressure, respectively. Root mean-square deviation (RMSD) and root mean-square fluctuation (RMSF) calculations per simulation frame that measured backbone flexibility (Cα atoms and amino-acid residues) were analyzed using CPPTRAJ (http://ambermd.org/tutorials/analysis/#cpptraj)^66^ and visualized in VMD (http://www.ks.uiuc.edu/Research/vmd/). Graphical representations of the RMSD and RMSF values were made using Graph Pad Prism software.

In silico analysis of domain motion in GG-SpaD was performed with the DynDom program^49^ (http://fizz.cmp.uea.ac.uk/dyndom/). Refined coordinates for the GG-SpaD_C_ and GG-SpaD_O_ crystal structures were uploaded onto the DynDom server and the analyses for conformational change were performed with default settings. Disorder predictions were initially done through the DISOPRED^55^ program on the XtalPred server (http://ffas.burnham.org/XtalPred-cgi/xtal.pl) using the sequence of GG-SpaD that was used in protein production. DISOPRED on the PSIPRED server (http://bioinf.cs.ucl.ac.uk/psipred) was later used to predict a disordered protein-binding site. A search for GG-SpaD-related sequences in the UniProt Reference Clusters database and their alignment was carried out using tools from the UniprotKB site (http://www.uniprot.org/). PISA (Protein Interfaces, Surfaces and Assemblies)^56^ at the European Bioinformatics Institute (http://www.ebi.ac.uk/pdbe/prot_int/pistart.html) was used to analyze the interaction and interface between the two GG-SpaD_C_ molecules in the crystal unit. Multiple protein structural alignments were performed using MUSTANG (http://lcb.infotech.monash.edu.au/mustang), with the corresponding sequence alignment diagram prepared by using Chimera (https://www.cgl.ucsf.edu/chimera).

Computational blind docking of the C-domain to the N-domain of GG-SpaD_C_ was done using the ClusPro^50^ web server (https://cluspro.bu.edu/). The N- and C-domains from the crystal structure of GG-SpaD_C_ were used as the receptor and ligand, respectively. Representative models from the top ten most favored clusters were predicted by different scoring modes (i.e., balanced, electrostatic-favored, hydrophobic-favored, and van der Waals + electrostatics-favored) and analyzed visually. The third and eighth models from the electrostatic-favored and balanced, respectively, scoring modes had resembled the docked molecules in the crystal structures.

### Immunoblotting of mutant SpaCBA-piliated lactococcal clones

As Lys180 of the YPKD pilin motif peptide in the N-domain of GG-SpaD is predicted to form an intermolecular K–T isopeptide bond, this was tested by making the corresponding K180A substitution in a nisin-inducible recombinant SpaFED-piliated *L. lactis* clone (GRS1189)^45^ and observing the effect on pilus assembly. In addition, Asp181 of the YPKD peptide was examined similarly (D181A), as this residue forms an internal isopeptide bond with Lys45, though this position is typically occupied by an asparagine in the more commonly found YPKN pilin motif. The construction of the mutant clones GRS1234 (K180A) and GRS1232 (D181A) was largely done as described previously^29,45^. For this, the expression plasmid (pKTH5393) containing the *L. rhamnosus* GG *spaFED* pilus genes acted as the template for adding the needed nucleotide changes (AAA → GCG for K180A or GAC → GCG for D181A) into the *spaD* gene by the overlap extension PCR technique^67^. Mutagenic PCR primers for the site-directed modifications were as follows: for K180A, forward 5'-CATCTTTATCCTGCGGACAGTCTTGTTAC-3' and reverse 5'-GTAACAAGACTGTCCGCAGGATAAAGATG-3' and for D181A, forward 5'-CATCTTTATCCTAAAGCGAGTCTTGTTAC-3' and reverse 5'-GTAACAAGACTCGCTTTAGGATAAAGATG-3'. Mutant plasmids pKTH5456 (for K180A) and pKTH5455 (for D181A) were introduced into *L. lactis* NZ9000 by standard electroporation^68^. Afterward, the K180A and D181A substitutions were corroborated by DNA sequencing. Growth and nisin-induced expression of lactococcal cells were carried out as done before^45^. Included as positive and negative controls were lactococcal clones GRS1189 and GRS1052 (containing empty-vector pKTH5080; unpublished), respectively. Immunoblotting experiments were performed using established methods. Briefly, cell pellets were recovered by centrifugation, and then washed (once) and resuspended with phosphate-buffered saline. Lactococcal cell suspensions were mixed with Laemmli buffer (1:1), incubated at 100 °C for at least 5 min, and later clarified centrifugally. Protein content was resolved electrophoretically on a precast 4–20% SDS-polyacrylamide gel (Bio-Rad) and followed by electroblotting onto a 0.45-μm nitrocellulose membrane (Bio-Rad). The membrane was then probed with primary (1:5000 diluted rabbit anti-GG-SpaD serum^58^) and secondary (1:10,000 diluted horseradish peroxidase-conjugated goat anti-rabbit IgG; Bio-Rad) antibodies. GG-SpaD bands were detected by using a chemiluminescent kit (Western Lightning Plus-ECL; Perkin Elmer, Inc.) according to manufacturer instructions.

### Data availability

The atomic coordinates of the models and their corresponding structure factors have been deposited in the Protein Data Bank (www.pdb.org) with the entry codes 5YU5 (WT full-length in closed/linear conformation), 5YXO (WT full-length in open/bent conformation), 5YXG (WT C-terminal fragment derived from limited proteolysis), 5Z0Z (D242A), and 5Z24 (K365A). All other data not present in the published paper or supplementary files are available from the authors upon reasonable request.

## Electronic supplementary material

Supplementary Information
