# Supplementary material for: Bent conformation of a backbone pilin N-terminal domain supports a three-stage pilus assembly mechanism
Source: Commun Biol. 2018 Jul 17;1:94. doi: 10.1038/s42003-018-0100-0 (PMC6123636; doi:10.1038/s42003-018-0100-0)
Supplement: Supplementary file 1 — Supplementary Information [file 42003_2018_100_MOESM1_ESM.pdf]

Supplementary Note 1. Structural comparison of GG-SpaD. Based on the superimposition with structures of other three-domain pilins (and two-domain GG-SpaA), GG-SpaD$_C$ is topologically similar but also distinctive, as certain differences are apparent (main text Fig. 8a-g). Here, GG-SpaD$_C$ does not exhibit metal sites (main text Fig. 8a), though the structures of all three-domain backbone pilins show the presence of a calcium-binding site (main text Fig. 8b-e). Moreover, GG-SpaD$_C$ shows structure for a portion of the C-terminal motif (i.e., LPXTG), which seems to be absent in other pilins, although it was part of the protein construct used to crystallize *S. pyogenes* T6[1]. In addition, GG-SpaD$_C$ appears to display a more buried interface between the N- and M-domains. Because an N-domain in the open conformation has not been reported for other backbone pilin structures, we sought to make comparisons with other N-domains and examine the isopeptide bonds and AB loops. Regarding possible isopeptide bonds among the full-length structures of the three-domain backbone pilins, a slow-forming one in the N-domain is only associated with *C. diphtheriae* SpaD[2] (main text Fig. 8c). Other backbone pilins such as *C. diphtheriae* SpaA[3] (main text Fig. 8d) and *S. pyogenes* T6[1] (main text Fig. 8e) lack the key residues for an isopeptide bond, and whereas *A. oris* FimP[4] possesses the necessary residues (main text Fig. 8b), the corresponding isopeptide bond is unformed. On the other hand, the GG-SpaD$_C$ structure contains an intact isopeptide bond in the N-domain (main text Fig. 2a, 8a), but then again this feature is not observed in GG-SpaD$_O$. In the same way, two-domain GG-SpaA also has an intact isopeptide bond in the CnaB fold of its N-domain (main text Fig. 8f, g), but seemingly this does not form when the connecting linker region bends in the absence of the C-domain[5] (main text Fig. 8h). As per the findings from the GG-SpaA crystal unit[5], the bent linker of one N-domain is able to insert into the groove formed by the AB loop of another N-domain, the outcome of which causes the AB loop taking on an ordered shape. This likely explains why we were earlier able to obtain the crystal structure of the flexible N-domain of GG-SpaA on its own, whereas for other types of backbone pilins it has been a great challenge, either alone or as part of a full-length protein[2,6,7,8,9,10,11]. Up till then, the only reported solution structure of an N-domain by itself, though containing an unformed isopeptide bond was for the four-domain *S. pneumoniae* RrgB backbone pilin[12].

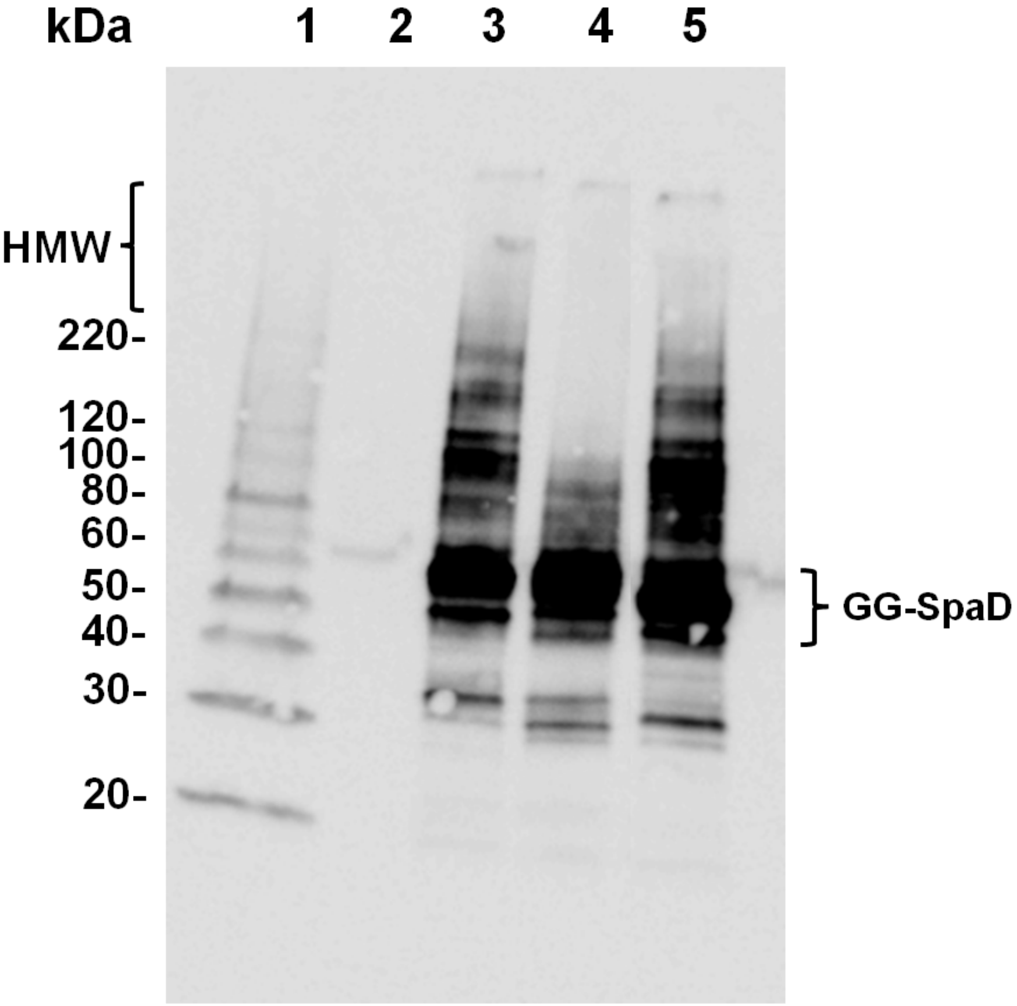

Supplementary Figure 1. The effect of GG-SpaD N-domain K180A and D181A substitutions on SpaFED pilus assembly. SpaFED pilus production from recombinant lactococcal cells is detected by immunoblotting with GG-SpaD-specific antiserum. The samples shown are for the empty vector GRS1052 (lane 2), SpaFED-piliated GRS1189 (lane 3), K180A-substituted GRS1234 (lane 4), and D181A-substituted GRS1232 (lane 5) lactococcal clones. SpaFED pili of various sizes are seen as a laddered pattern of protein bands, with the lengthiest pili appearing as compressed high-molecular-weight (HMW) protein bands. Monomeric GG-SpaD protein and molecular weight markers (lane 1) are identified to the right and left, respectively, of the immunoblot. Results from this immunoblot clearly show that Lys180 is needed for assembling GG-SpaD subunits into the pilus backbone structure, as evidenced by the absence of produced SpaFED pili from the K180A lactococcal mutant. Alternatively, the D181A substitution in the N-domain, which presumably abrogated the internal K-D isopeptide bond, had no observable effect on SpaFED pilus assembly. See main text Methods for detailed protocols.

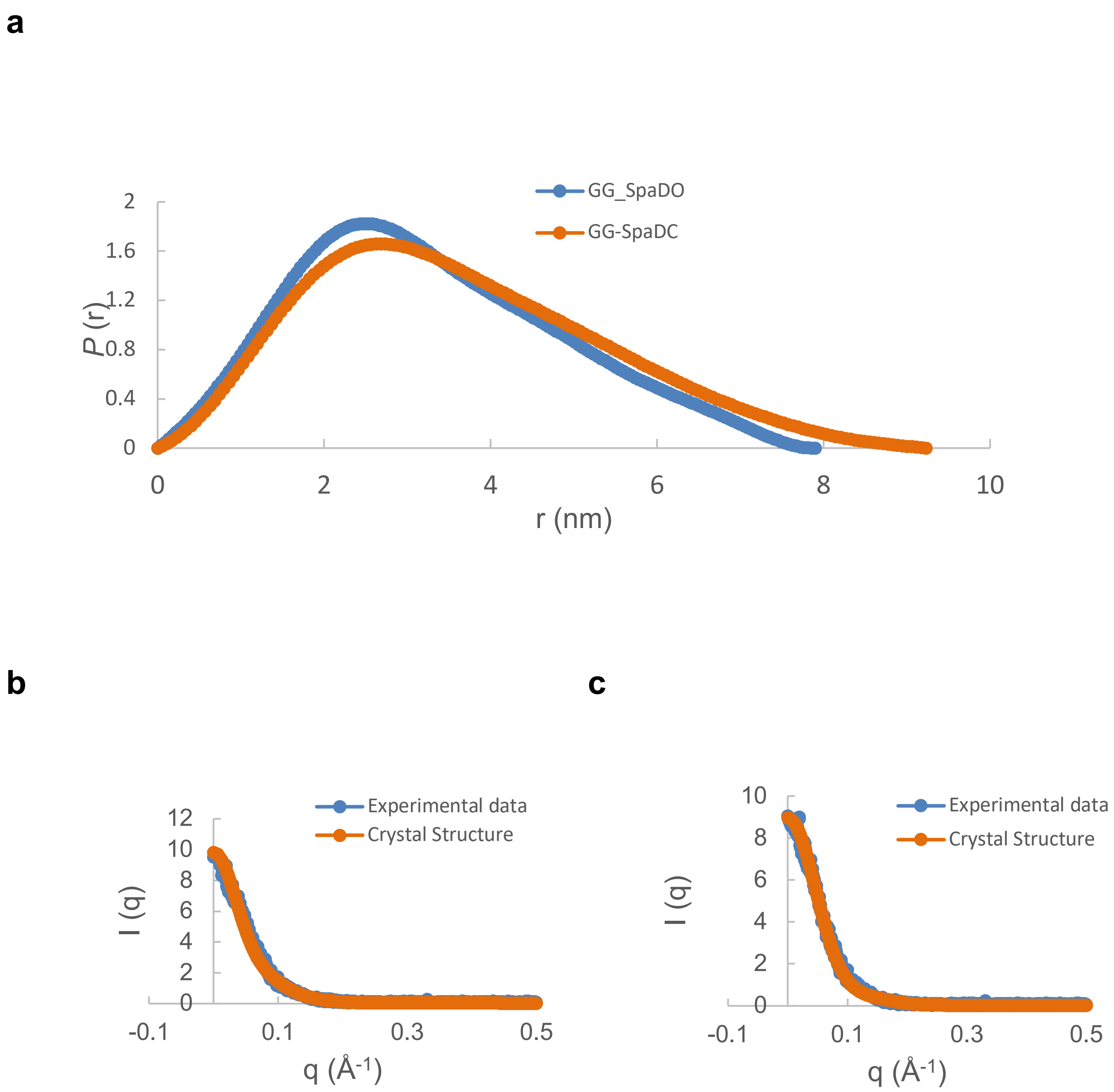

Supplementary Figure 2. Small angle X-ray scattering (SAXS) analysis of GG-SpaD$_C$ and GG-SpaD$_O$. a The pairwise interatomic distance distribution derived from SAXS for GG-SpaD$_C$ and GG-SpaD$_O$. The maximum distance (D_{max}) from *P*(r) for GG-SpaD$_C$ and GG-SpaD$_O$ is 9.23 and 7.92 nm, respectively. **b** Plot of log intensity of the scattering of GG-SpaD$_C$. The theoretical scattering curve (orange) calculated from the crystal structure was fitted to the experimental curve (blue) using CRYSOL. **c** Comparison of the SAXS profile (blue) and the theoretical scattering curve (orange) calculated from crystal structure for GG-SpaD$_O$.

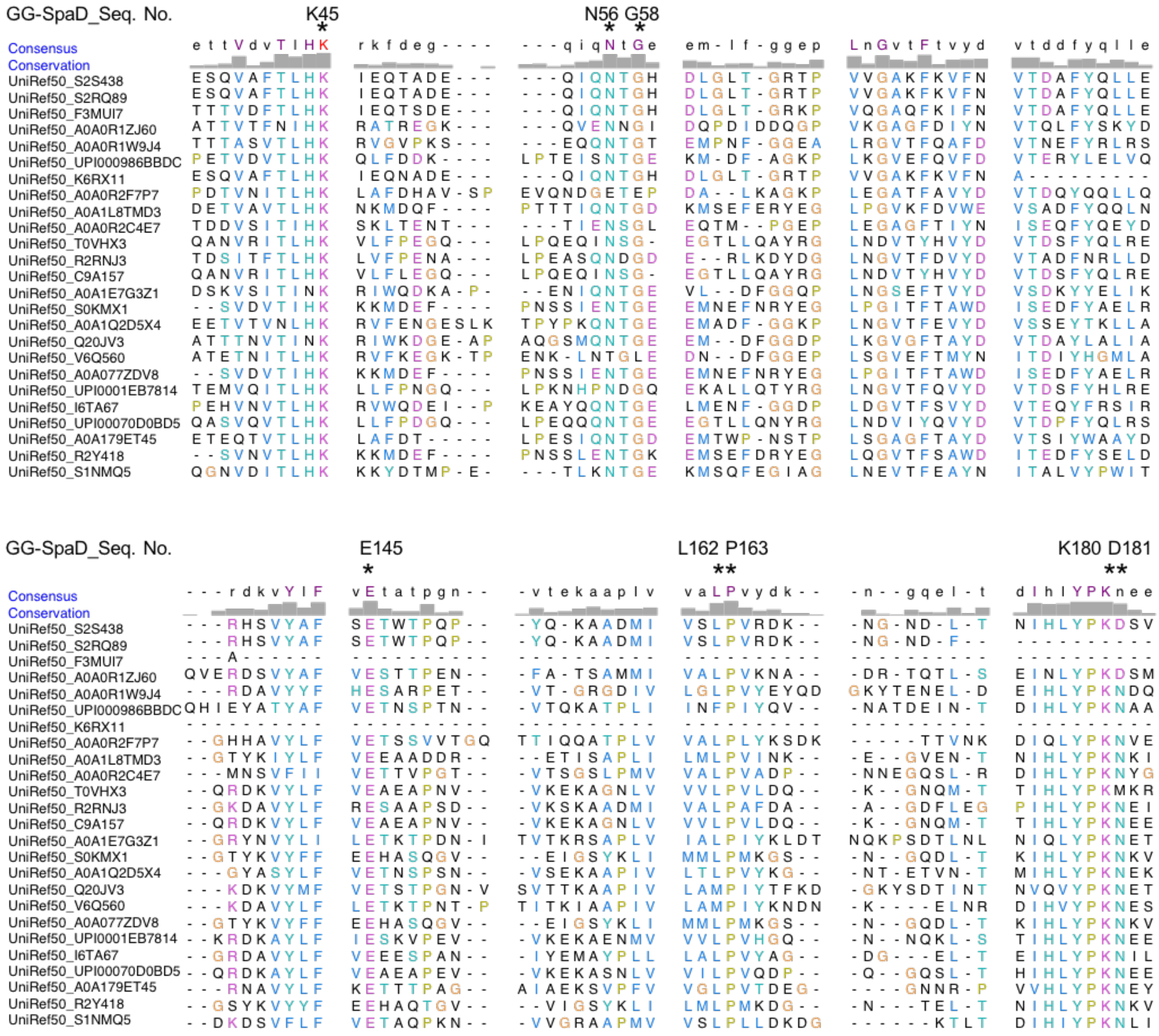

Supplementary Figure 3. Multiple sequence alignment of the N-domain sequences from GG-SpaD and its related homologs. Clustered sets of sequences for GG-SpaD and its related homologs are from the UniProt Reference Clusters (UniRef50). Cluster IDs are indicated to the left. Conserved segments (NTG and LP) within loop regions and those residues involved in isopeptide bond formation are identified at the top by an asterisk (*) and denoted according to the residue number of GG-SpaD. For clarity, only the N- and C-terminal regions of the structural N-domain are shown in the alignment.

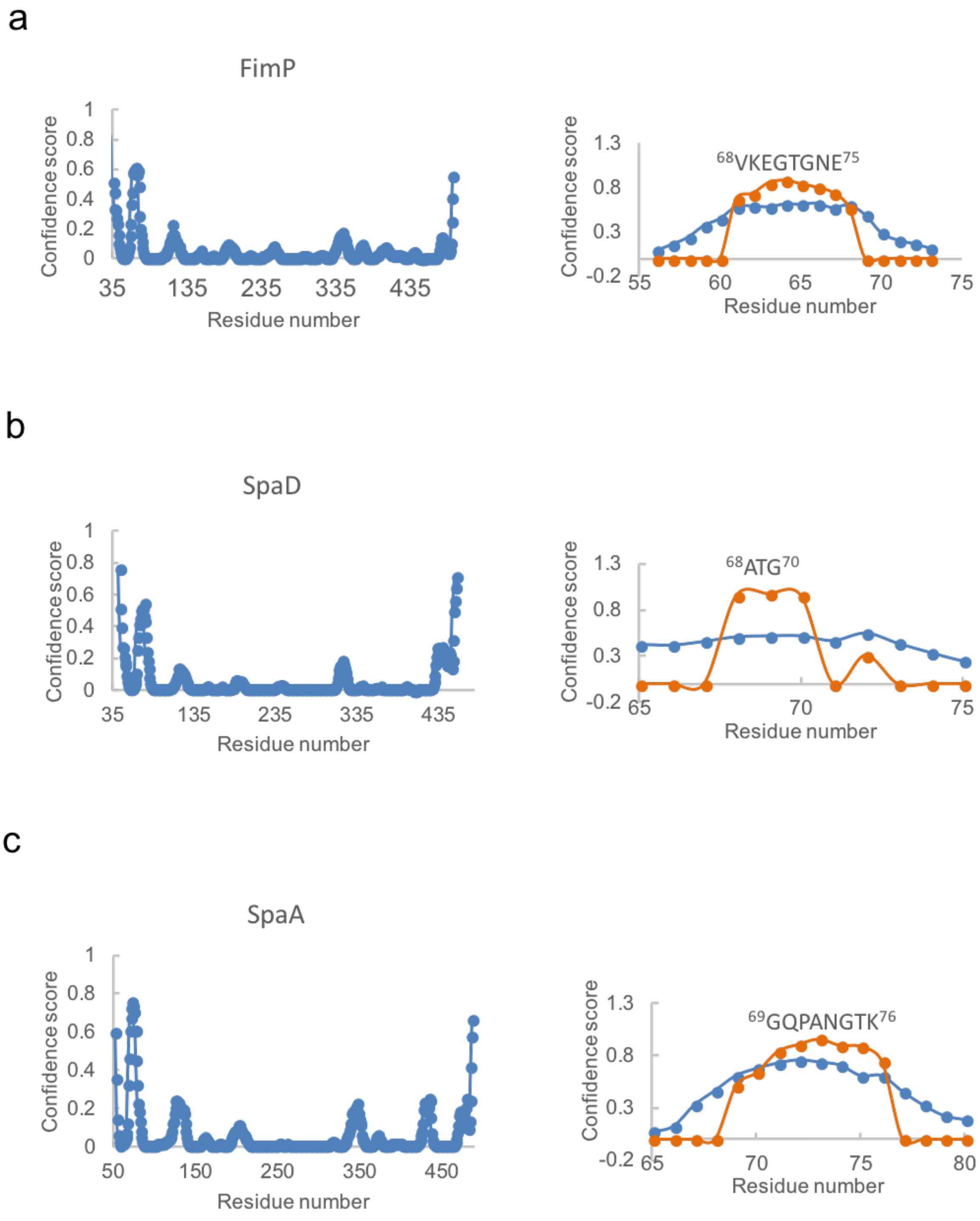
a
FimP
Confidence score
Residue number
68VKEGTGNE75
b
SpaD
Confidence score
Residue number
68ATG70
c
SpaA
Confidence score
Residue number
69GQPANGTK76

d

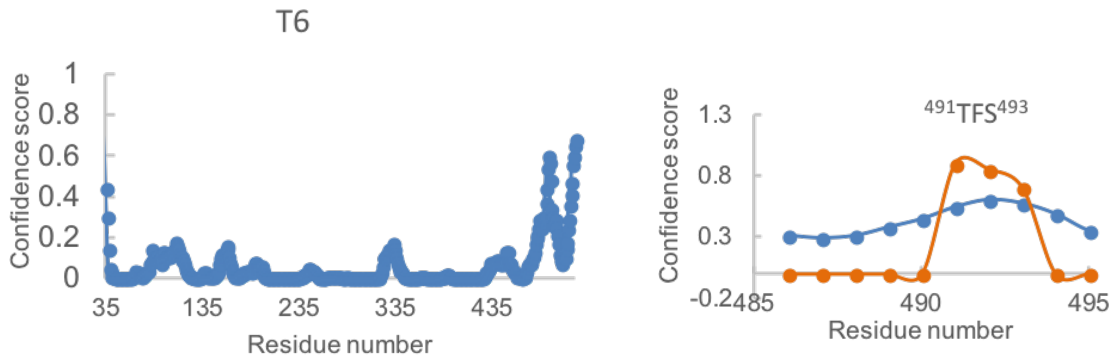

e

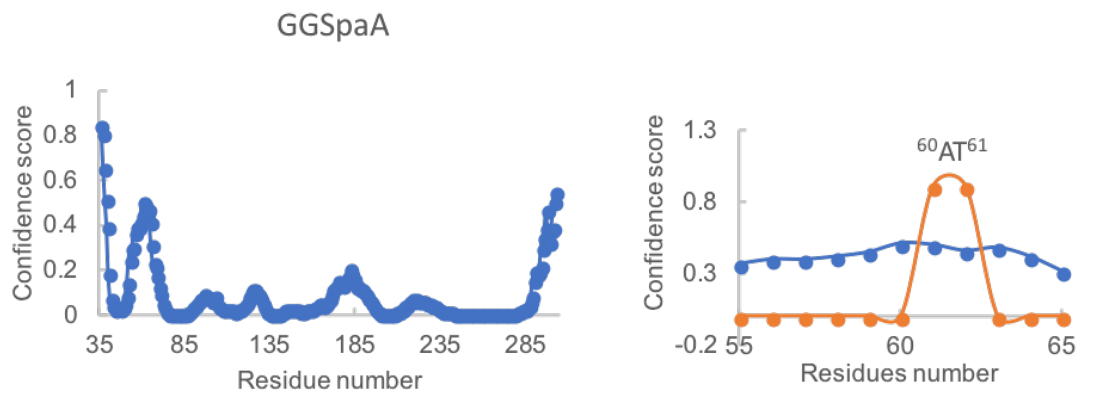

Supplementary Figure 4. Prediction of intrinsically disordered regions in GG-SpaD-related backbone pilins. Levels of disorder in *A. oris* FimP (**a**), *C. diphtheriae* SpaD (**b**), *C. diphtheriae* SpaA (**c**), *S. pyogenes* T6 (**d**), and GG-SpaA (**e**) were predicted using the DISOPRED program. Predicted regions in the primary structure reflecting disorder (blue) and a disordered protein-binding site (orange) are shown.

Supplementary Table 1. Oligonucleotide primers used for site-directed mutagenesis of GG-SpaD. Nucleotide changes for alanine substitutions are indicated in blue font.

Primer	Nucleotide sequence
**E145A forward**	5'-AGCGTGTACGCATTTCACGCAGCCGTGACCCCGCAACCG
**E145A reverse**	5'-CGGTTGCGGGGTCACGGCTGCGTGAAATGCGTACACGCT
**D242A forward**	5'-AACCAATTTAAAGTGCTGGCTTATATGACCAAGGAAGGC
**D242A reverse**	5'-GCCTTCCTTGGTCATATAAGCCAGCACTTTAAATTGGTT
**E445A forward**	5'-GGGCGATATACCTTGGTTGCAACCGCGGCACCAGAAGGC
**E445A reverse**	5'-GCCTTCTGGTGCCGCGGTTGCAACCAAGGTATATCGCCC
**K45A forward**	5'-GTGGATTTCACGCTGCACGCAATCGAACAAACCAGTGAC
**K45A reverse**	5'-GTCACTGGTTTGTTCGATTGCGTGCAGCGTGAAATCCAC
**K186A forward**	5'-AAAGACAGTCTTGTTACCGCAAATCTGACGGAAATCAAT
**K186A reverse**	5'-ATTGATTTCCGTCAGATTTGCGGTAACAAGACTGTCTTT
**K365A forward**	5'-GGCGGGATTCGGTTCTTCGCACACGAAGCCGGATCTTCC
**K365A reverse**	5'-GGAAGATCCGGCTTCGTGTGCGAAGAACCGAATCCCGCC

Supplementary Table 2. SAXS data measurements of GG-SpaD$_C$ and GG-SpaD$_O$.

**SAXS parameters**	**GG-SpaD**	
Instrument	SAXSess Anton Paar	
Beam Geometry	10 mm slit	
Wavelength (Å)	1.5418	
q range ($A^{\circ -1}$)	0.10–3.00	
Exposure time (min)	30 min	
Concentration (mg ml^{-1})	17.0	
Temperature (K)	283	
**Software employed**		
Primary data reduction	SAXS quant	
Data processing	PRIMUS QT	
Ab-initio analysis	DAMMIF	
Validation and averaging	DAMAVER	
Three-dimensional graphics	PyMOL	
**Structural parameters**	**GG-SpaD$_C$**	**GG-SpaD$_O$**
I(0) from Guinier (nm)	90853.20	87754.5
Rg from Guinier (nm)	2.81	2.56
I(0) from Pr (nm)	0.90143	0.87854
Rg from Pr (nm)	2.815	2.55
Dmax from Pr (nm)	9.23	7.92
Porod volume estimate ($Å^3$)	62.39	53.61

Supplementary Table 3. Domain motion analysis of the closed and open conformation structures of GG-SpaD as determined by DynDom.

DynDom parameters	GG-SpaD
Fixed domain	Residues 184-479 (RMSD-0.56 Å)
Moving domain	Residues 39-183 (RMSD-1.53 Å)
Rotation angle (°)	107.0
Translation (Å)	-2.5
Closure (%)	76.5
Bending residues	179-186

Supplementary Table 4. Interaction analysis of the two GG-SpaD molecules in the crystal lattice. Met486 and Thr487 were modeled in for interaction analysis, as these residues were excluded from the recombinant construct of GG-SpaD.

Sl. No.	Molecule 1	Distance	Molecule 2
**Hydrogen bonds**			
**1**	B:GLN 482 [NE2]	2.8	A:PRO 163 [O]
**2**	B:LEU 483 [N]	3.3	A:SER 161 [O]
**3**	B:THR 487 [OG1]	2.7	A:ILE 54 [O]
**4**	B:ALA 368 [O]	2.8	A:ARG 137 [NE]
**5**	B:GLY 369 [O]	3.6	A:ARG 137 [NH2]
**6**	B:PRO 479 [O]	3.4	A:TYR 84 [OH]
**7**	B:ASN 481 [OD1]	3.6	A:TYR 84 [OH]
**8**	B:ASN 481 [O]	2.7	A:SER 161 [OG]
**9**	B:GLN 482 [O]	3.1	A:LYS 92 [NZ]
**10**	B:LEU 483 [O]	3.0	A:SER 161 [N]
**11**	B:LEU 484 [O]	3.7	A:GLN 55 [NE2]
**12**	B:PRO 485 [O]	3.2	A:GLN 55 [NE2]
**13**	B:PRO 485 [O]	3.5	A:ASN 56 [N]
**14**	B:THR 487 [OG1]	3.1	A:ASN 56 [N]
**15**	B:THR 487 [O]	3.2	A:GLN 53 [NE2]
**16**	B:THR 487 [O]	3.5	A:ILE 54 [N]
**Salt bridges**			
**1**	B:GLU 367 [OE2]	3.5	A:ARG 137 [NH2]
**2**	B:GLU 367 [OE2]	3.9	A:ARG 137 [NE]
**3**	B:GLU 453 [OE1]	3.5	A:ARG 165 [NH2]
**4**	B:GLU 453 [OE1]	2.8	A:ARG 165 [NH1]
**5**	B:GLU 453 [OE1]	3.6	A:ARG 165 [NH2]
**Residue with most stabilizing effect**			
			A:SER 161
			A:ASN 56
			A:TYR 84

Supplementary Table 5. DALI search for structural homologs of GG-SpaD. Top five hits are listed.

PDB code	Name	Pilin type	Bacterial host source	Z-score	RMSD (Å)	No. of aligned residues	Sequence identity (%)
**Full-length (Closed): GG-SpaD$_C$**							
3UXF	FimP	backbone	*A. oris*	18.0	5.5	361	21
4HSS	SpaD	backbone	*C. diphtheriae*	18.0	5.7	341	19
3HTL	SpaA	backbone	*C. diphtheriae*	17.1	6.8	342	19
3PF2	GBS80	backbone	*S. agalactiae*	17.0	3.1	245	23
4UZG	Bp-2b	backbone	*S. agalactiae*	16.7	3.9	234	21
5HBB	GG-SpaA	backbone	*L. rhamnosus* GG	15.9	1.8	114	25
**Full-length (Open): GG-SpaD$_O$**							
2XTL	Bp-2a	backbone	*S. agalactiae*	19.1	2.6	246	26
3PF2	GBS80	backbone	*S. agalactiae*	18.5	3.0	242	23
4UZG	Bp-2b	backbone	*S. agalactiae*	18.3	3.8	231	21
2Y1V	RrgB	backbone	*S. pneumoniae*	16.3	2.8	249	26
4HSS	SpaD	backbone	*C. diphtheriae*	15.6	10.4	246	19
**N-domain**							
4HSS	SpaD	backbone	*C. diphtheriae*	14.3	2.8	126	24
3UXF	FimP	backbone	*A. oris*	13.2	2.5	123	29
3PHS	GBS52	basal	*S. agalactiae*	11.0	2.3	106	16
3HTL	SpaA	backbone	*C. diphtheriae*	10.5	2.8	117	19
3KPT	BcpA	backbone	*B. cereus*	9.9	2.2	92	20
**M-domain**							
2XTL	Bp-2a	backbone	*S. agalactiae*	14.2	2.1	136	24
2Y1V	RrgB	backbone	*S. pneumoniae*	13.8	2.5	135	27
4TSH	P1	adhesin	*S. mutans*	13.3	2.8	151	15
3QE5	AgI/II	Antigen	*S. mutans*	13.1	2.8	150	15
3KPT	BcpA	backbone	*B. cereus*	13.0	2.4	132	14
**C-domain**							
5HBB	GG-SpaA	backbone	*L. rhamnosus* GG	15.6	1.8	114	25
4UZG	Bp-2b	backbone	*S. agalactiae*	14.5	1.8	105	26
2XTL	BP-2a	backbone	*S. agalactiae*	13.1	2.2	110	27
4OQ1	RrgC	tip	*S. pneumoniae*	13.0	2.1	106	20
3PHS	GBS52	basal	*S. agalactiae*	12.9	2.2	102	25

Supplementary Table 6. Crystallization conditions for GG-SpaD proteins. All crystallization experiments were performed at room temperature (295K) using the hanging drop-vapor diffusion method by equilibrating a 2-μl drop (1 μl protein and 1 μl reservoir solution) against a 1-ml volume of reservoir solution.

	Protein buffer	Protein concentration (mg ml^{-1})	Reservoir solution	Additives	Cryoprotectant
**GG-SpaD$_C$**	20mM HEPES pH7.5, 150mM NaCl,1mM EDTA	50	0.1M MES pH6.5, 30% (v/v) PEG400	None	20% (v/v) PEG 400
**GG-SpaD$_O$**	20mM HEPES pH7.5, 150mM NaCl, 1mM EDTA	16	30% (w/v) PEG1500	1M NaCl	20% (v/v) PEG 400
**GG-SpaD$_{MC}$**	20mM HEPES pH7.5, 150mM NaCl	35	0.17M Lithium sulphate, 25% (w/v) PEG3350	0.1M Strontium chloride hexahydrate	30% (v/v) Ethylene glycol
**GG-SpaD$_{SeMet}$**	20mM HEPES pH7.5, 150mM NaCl	45	0.2M Ammonium acetate, 0.1M MES pH6.5, 30% (v/v) PEG 400	None	20% (v/v) PEG 400
**GG-SpaD$_{D242A}$**	20mM HEPES pH7.5, 150mM NaCl	50	0.2M Magnesium acetate, 10% (w/v) PEG8000	None	0.6M Magnesium acetate tetra hydrate
**GG-SpaD$_{K365A}$**	20mM HEPES pH7.5, 150mM NaCl	45	0.2M Magnesium acetate, 0.1M HEPES pH7.5, 15% (v/v) PEG400	None	1M Lithium acetate

Supplementary Table 7. Ramachandran statistics for the refined structures of GG-SpaD.

	GG-SapD$_{C}$	GG-SpaD$_{O}$	GG-SpaD$_{MC}$	GG-SpaD$_{D242A}$	GG-SpaD$_{K365A}$
**Favored (%)**	97.96	96.31	98.39	96.85	96.14
**Allowed (%)**	2.04	3.69	1.61	3.15	3.86
**Outliers (%)**	0	0	0	0	0

Supplementary Table 8. Data collection and phasing statistics for the GG-SpaD$_{SeMet}$. Each dataset was collected from a single crystal. Data values in parentheses are for the highest-resolution shell.

Parameters	Peak	Remote	Inflection
**Data Collection**			
Space group	*P* 2_1 2_1 2_1		
**Cell dimensions**			
a, b, c (Å)	47.27, 70.22, 395.63	47.80, 70.77, 395.78	47.60, 70.46, 395.14
α, β, γ (°)	90, 90, 90	90, 90, 90	90, 90, 90
Wavelength (Å)	0.97887	0.95372	0.9791
Resolution range (Å)	42.65-2.81 (2.96-2.81)	44.17-2.82 (2.97-2.82)	44.05-2.82 (2.97-2.82)
R_{merge}	0.10 (0.76)	0.15 (1.36)	0.13 (1.13)
I/σ(I)	18.0 (3.7)	18.6 (2.9)	17.7 (2.7)
Completeness (%)	99.9 (99.8)	99.9 (99.9)	99.9 (99.9)
No. reflections	33584 (4788)	33685 (4804)	33348 (4738)
Redundancy	14.2 (14.7)	14.5 (14.9)	14.4 (14.9)
$CC_{1/2}$	0.99 (0.98)	0.99 (0.94)	0.99 (0.94)
DelAnom correlation between half-sets	0.54 (0.03)	0.24 (0.01)	0.42 (0.02)
Mid-slope of anom normal probability	1.041	1.062	1.075
**Phasing statistics**			
No. of selenium sites	16		
Phasing power anomalous	1.007	0.567	0.724
Phasing power isomorphous (acentric/centric)		0.272/0.254	0.190/0.170
R_{cullis} anomalous	0.85	0.93	0.91
R_{cullis} isomorphous (acentric/centric)		0.56/0.59	0.49/0.53
Overall figure of metric (acentric/centric)	0.293/0.146		

Supplementary References.

1. Young, P. G., *et al.* Structural conservation, variability, and immunogenicity of the T6 backbone pilin of serotype M6 *Streptococcus pyogenes*. *Infect. Immun.* **82**, 2949-2957 (2014).
2. Kang, H. J., *et al.* A slow-forming isopeptide bond in the structure of the major pilin SpaD from *Corynebacterium diphtheriae* has implications for pilus assembly. *Acta Crystallogr. D Biol. Crystallogr.* **70**, 1190-1201 (2014).
3. Kang, H. J., Paterson N. G., Gaspar A. H., Ton-That H. & Baker E. N. The *Corynebacterium diphtheriae* shaft pilin SpaA is built of tandem Ig-like modules with stabilizing isopeptide and disulfide bonds. *Proc. Natl. Acad. Sci. USA* **106**, 16967-16971 (2009).
4. Persson, K., Esberg A., Claesson R. & Stromberg N. The pilin protein FimP from *Actinomyces oris*: Crystal structure and sequence analyses. *PLoS One* **7**, e48364 (2012).
5. Chaurasia, P., Pratap S., von Ossowski I., Palva A. & Krishnan V. New insights about pilus formation in gut-adapted *Lactobacillus rhamnosus* GG from the crystal structure of the SpaA backbone-pilin subunit. *Sci. Rep.* **6**, 28664 (2016).
6. Nuccitelli, A., *et al.* Structure-based approach to rationally design a chimeric protein for an effective vaccine against Group B Streptococcus infections. *Proc. Natl. Acad. Sci. USA* **108**, 10278-10283 (2011).
7. Cozzi, R., *et al.* Structure and assembly of group B Streptococcus pilus 2b backbone protein. *PLoS One* **10**, e0125875 (2015).
8. Mishra, A., *et al.* Two autonomous structural modules in the fimbrial shaft adhesin FimA mediate Actinomyces interactions with streptococci and host cells during oral biofilm development. *Mol. Microbiol.* **81**, 1205-1220 (2011).
9. Spraggon, G., *et al.* Supramolecular organization of the repetitive backbone unit of the *Streptococcus pneumoniae* pilus. *PLoS One* **5**, e10919 (2010).
10. Vengadesan, K., Ma X., Dwivedi P., Ton-That H. & Narayana S. V. A model for group B Streptococcus pilus type 1: the structure of a 35-kDa C-terminal fragment of the major pilin GBS80. *J. Mol. Biol.* **407**, 731-743 (2011).
11. Budzik, J. M., *et al.* Amide bonds assemble pili on the surface of bacilli. *Proc. Natl. Acad. Sci. USA* **105**, 10215-10220 (2008).
12. Gentile, M. A., *et al.* Structural and functional characterization of the *Streptococcus pneumoniae* RrgB pilus backbone D1 domain. *J. Biol. Chem.* **286**, 14588-14597 (2011).
